# Glucocorticoid measurement in plasma, urates, and feathers from California condors (*Gymnogyps californianus*) in response to a human-induced stressor

**DOI:** 10.1371/journal.pone.0205565

**Published:** 2018-10-23

**Authors:** Zeka E. Glucs, Donald R. Smith, Christopher W. Tubbs, Jennie Jones Scherbinski, Alacia Welch, Joseph Burnett, Michael Clark, Curtis Eng, Myra E. Finkelstein

**Affiliations:** 1 Microbiology and Environmental Toxicology Department, University of California, Santa Cruz, CA, United States of America; 2 San Diego Zoo Global, Institute for Conservation Research, Escondido, CA, United States of America; 3 National Park Service, Pinnacles National Park, Paicines, CA, United States of America; 4 Ventana Wildlife Society, Monterey, CA, United States of America; 5 Los Angeles Zoo and Botanical Gardens, Los Angeles, CA, United States of America; Universite de Rouen, FRANCE

## Abstract

Vertebrates respond to stressful stimuli with the secretion of glucocorticoid (GC) hormones, such as corticosterone (CORT), and measurements of these hormones in wild species can provide insight into physiological responses to environmental and human-induced stressors. California condors (*Gymnogyps californianus*) are a critically endangered and intensively managed avian species for which information on GC response to stress is lacking. Here we evaluated a commercially available I^125^ double antibody radioimmunoassay (RIA) and an enzyme-linked immunosorbent assay (ELISA) kit for measurement of CORT and GC metabolites (GCM) in California condor plasma, urate, and feather samples. The precision and accuracy of the RIA assay outperformed the ELISA for CORT and GCM measurements, and CORT and GCM values were not comparable between the two assays for any sample type. RIA measurements of total CORT in condor plasma collected from 41 condors within 15 minutes of a handling stressor were highly variable (median = 70 ng/mL, range = 1–189 ng/mL) and significantly different between wild and captive condors (p = 0.02, two-tailed t-test, n = 10 wild and 11 captive). Urate GCM levels (median = 620 ng/g dry wt., range = 0.74–7200 ng/g dry wt., n = 216) significantly increased within 2 hr of the acute handling stressor (p = 0.032, n = 11 condors, one-tailed paired t-test), while feather section CORT concentrations (median = 18 pg/mm, range = 6.3–68 ng/g, n = 37) also varied widely within and between feathers. Comparison of multiple regression linear models shows condor age as a significant predictors of plasma CORT levels, while age, sex, and plasma CORT levels predicted GCM levels in urates collected within 30 min of the start of handling. Our findings highlight the need for validation when selecting an immunoassay for use with a new species, and suggest that non-invasively collected urates and feathers hold promise for assessing condor responses to acute or chronic environmental and human-induced stressors.

## Introduction

When used appropriately, assessment of glucocorticoid (GC) hormone levels in wildlife species can be a meaningful indicator of physiological stress and ability to respond to energetic demands of their environment [1–4]. The time of sample collection in relationship to a known stressor informs interpretation of GC data in wild animals. Measurement of circulating GC levels prior to a stressor has been interpreted as the allostatic load or “baseline” physiological GC requirement [5,6], and is often subject to seasonal and diel modulation due to predictable differences in energy availability and energy requirements [7,8]. Measurements of elevated GC after a known stressor provide information on responsiveness of the GC secretion pathway, which can also be impacted by chronic stress [9]. When the experimental stressor is not precisely controlled, individual plasma GC measurements are difficult to interpret as they represent a single time point within the hormone stress response that typically occurs over hours to days [10].

In light of these issues, plasma GC measurements are not always appropriate or feasible for GC stress response comparisons in field settings. Methods for GC quantification in other sample types such as feces, urates, fur, and feathers have been developed for assessing stress status in wild animals [11,12]. Measurements of GCs and GC metabolites (GCM) in feather and urates provide a means for assessing baseline or elevated GC levels in wild condors because of the longer time lag between stressor and hormone elevation, as in urates, or the ability to retrospectively assess circulating GC levels integrated over days to months, as in feathers [13].

The development of enzyme-linked immunosorbent assay (ELISA) kits for hormone detection has made GC measurements more accessible, as ELISA methods obviate the need for specialized laboratory equipment required for measurement of radioactive materials in traditional radioimmunoassay (RIA)-based methods, or the need for radioactive waste disposal [13]. Many immunoassay kits are optimized for detecting parent hormones in mammalian plasma, although manufacturers sometimes advertise kit compatibility with other biological samples such as saliva and excreta (e.g. Corticosterone ELISA, Enzo Life Sciences). Immunoassay kits have been validated for GC and GCM measurement in a variety of non-plasma sample types in birds (in feces [14,15]; in feathers [16]). However, metabolic pathways for GCs and sample matrix composition can be both species- and sample-specific, and the antibodies in each immunoassay kit may interact with sample GC differently depending on GCMs present or matrix composition of the sample [17]. Significant inter-laboratory variation in GC measurement in avian plasma has also been reported [18]. As such, analytical validation of immunological GC and GCM measurement methods for the specific target species and sample type (e.g., plasma, feces, urates, etc.) is needed before embarking on studies to assess GC and GCM concentrations as an indicator of physiological stress [11,13,19,20].

The California condor (*Gymnogyps californianus*) is a critically endangered vulture for which no GC measurements have been reported to our knowledge. Wild condors undoubtedly experience a variety of stressors, such as frequent lead poisoning [21], and semi-annual capture and handling for health monitoring, tracking equipment maintenance, and clinical intervention for lead poisoning events if warranted [22]. Direct physiological measurements are necessary for assessing individual condor health as the population scale metrics often used for wildlife studies, such as survival and reproductive rates, are manipulated by endangered species management protocols [22]. The natural, lead, and management-related stressors experienced by condors may prove deleterious to their health and survival and underscore the pressing need for assessing California condor GC measurements. Collecting pre-stressor plasma is difficult if not impossible in wild avian species such as the condor that are very large and difficult to trap and handle. The process of capturing condors within a flight pen and performing a blood draw can take tens of minutes, but circulating GCs in other avian species have been found to elevate within 2–3 minutes of a handling stressor [23]. Therefore, collection of peripheral samples such as urates and feather for GC measurement could enable researchers to assess GC status in condors trapped from the wild.

In this study, we identify an appropriate method to compare the condor GC stress-response among tissues and between individual condors, and investigate the potential influence of biological factors (i.e. age, sex, and season) and existing California condor trapping protocols on GC release in this species. We evaluated the precision and accuracy of a competitive corticosterone (CORT) enzyme-linked immunosorbent assay (ELISA) (Cat. No. ADI-900-097, Enzo Life Sciences), and a corticosterone ^125^I double antibody radioimmunoassay (RIA) kit (Prod. No. 07120103, MP Biomedicals) for measurement of plasma and feather CORT concentrations and urate GCM concentrations in California condors. The endangered status of the California condor contraindicated pharmacological challenges used in some species to test GC responsivity, such as adrenocorticotropic hormone or dexamethasone injections. In light of this constraint, we performed a biological method validation using handling and restraint as the acute stressor, as recommended by Touma and Palme [20] and employed in other field studies [24]. We present our method validation and in depth examination of the factors influencing GC levels as a framework for wildlife biologists preparing to measure and interpret GCs in a previously unstudied, free-ranging species, or hoping to use a single immunoassay for GC measurement across multiple sample types.

## Materials and methods

### Study subjects

Wild condors (n = 41) from central California, managed by Pinnacles National Park and Ventana Wildlife Society, and captive condors (n = 11) housed at the Los Angeles Zoo and Botanical Gardens and Santa Barbara Zoo were sampled for plasma and urates between 2013 and 2016, and feathers between 2008 and 2010 (S1 Table). Both male and female condors were included, with ages ranging 1–36 years (S1 Table). The use of vertebrate samples for this research was approved by The University of California Santa Cruz’s Institutional Animal Care and Use Committee with permission from Los Angeles Zoo and Botanical Gardens and Santa Barbara Zoo (IACUC office approval code FINKM1307). Samples were collected at Pinnacles National Park and Ventana Wildlife Society under USFWS sub-permits (Pinnacles National Park Permit # TE 157291–1; Ventana Wildlife Society Permit # TE-026659-14).

### Stress challenge and sample collections

Plasma, urate, and feather samples were collected during scheduled handling events for routine health monitoring as part of condor management protocols [22]. Wild condors were passively captured using carcass-baited double door traps, operated by technicians concealed within a blind to prevent detection of human presence at the trap. Birds were then given access through another blind-operated door to a larger, netted, flight pen (dimensions ~7.5 m x 12 m x 6 m tall). Birds were typically held in the larger flight pen for >24 hr (range = 19–223 hr, median = 45 hr) before handling commenced. Captive birds were housed permanently in flight pens of similar dimensions and design. Condors in flight pens were normally housed in groups of 2–9 individuals and had access to food and water *ad libitum*, or in the case of captive individuals, access to food on a weekly basis with set fast days to mimic condor feeding behavior in the wild. While in the flight pen, condors were visually isolated from humans until the day of handling.

On the day of handling, the time of initial pen entry by technicians was recorded for each handling event (median = 61 min, range = 10–200). Following pen entry by technicians, condors were herded either onto the ground or into enclosed isolation pens, a process that can take 2–15 min. Handling start was recorded upon a bird’s capture by a large, hand-held hoop net. The condor was then restrained in hand for a median time of 27 min (range = 14–47 min) for blood draw, possible feather collection, physical examinations, and in the case of wild birds, tracking equipment maintenance. Information on body condition (keel ratings and hydration status) were collected for some but not all individuals during these exams (S1 Table). Keel ratings were collected by feel and hydration status was collected by visual observation of skin elasticity after pinching, and therefore both were subject to variation based on technician. To minimize technician-related bias, we coded these categorical observations as binary (Keel status: 0 = breast muscle concave to keel, 1 = breast muscle even with or convex to keel; Hydration status: 0 = dehydrated, 1 = well hydrated).

#### Plasma collection

Blood samples were collected within 3–18 min of handling start (median = 6 min). Blood (1–2 mL) was collected from the metatarsal vein into heparinized vacutainers and placed on ice. Plasma was separated from whole blood via centrifugation (10 min at 2,000 x g) within 12 hr of blood collection, transferred into cryovials, and stored at -80°C until analysis.

#### Urate collection

Immediately after handling for routine health monitoring as described above, condors were placed into a modified dog kennel similar to those used for condor transport, but altered with a raised vinyl-coated mesh floor above a removable Plexiglas tray to collect urate and fecal samples. The environment inside the kennel was low light and visually isolated from outside stimuli. The kennel had sufficient room to allow the condor to turn around and lay in repose. Wild birds were held within kennels for 1–6 hr before release to the wild or transport to clinics for lead poisoning or other medical treatment if warranted, which was as long as was logistically feasible. Condors did not have access to food and water while in the kennel. Urate and fecal samples were serially collected from the sub-floor tray which was checked at 15 min intervals while birds were kenneled. However, if urate excretion was detected (by sound) before the 15 min tray check, it was collected immediately. Urates were placed on dry ice within 30 min of defecation and stored at -80°C until extraction to arrest hormone metabolism by bacterial enzymes [11,20].

As with other new world vulture species, California condors perform urohidrosis, and generally excrete fecal (i.e. solid) and urate (i.e. liquid) material separately [25]. Since urates were collected more consistently and frequently during condor kenneling time periods, we chose to use GCM levels in urates for comparison across individuals in this study. In the few cases when urates and feces were excreted together, we collected the dark solid excrement and white-clear liquid urate excrement separately. In order to examine if there were differences across urate samples with respect to hydration and potential fecal mixing, we recorded a color code of 1–5 for each urate sample collected (1 = white/clear, 2 = white/yellow, 3 = yellow, 4 = some green, 5 = green/brown).

#### Feather collection

We utilized previously collected feather samples, as the complete trailing edge of flight feather vane (base to tip) is regularly collected during handling events as part of lead exposure monitoring studies[26–28]. Feather vane material from growing and nearly full-grown flight feathers was either collected at the time of handling, or if only partially grown, flight feathers were marked via notching, measured from the base of the feather (at skin level) to tip, and later sampled at a subsequent handling event when the feather was fully grown (S2 Table). Feather samples were stored at room temperature in plastic bags in low light.

### Sample processing and hormone extraction

#### Plasma processing

Prior to analysis, plasma samples were thawed, vortexed and diluted with kit buffers in accordance with kit instructions, with the exception of using half volumes of all samples and kit reagents in the MP Biomedicals radioimmunoassay as described and validated for avian plasma in Washburn et al. [29].

#### Urates processing and extraction

Whole urate samples were lyophilized and re-suspended in 80% methanol (Certified ACS, A412-4, Fisher Scientific) at a ratio of 75 mL:1 g dry weight; because of differences in urate sample weights, a range of 1–32 mL methanol was added per sample (adapted from methods [30–32]). The sample-solvent mixture was vortexed for 15 sec, shaken at room temperature for 30 min on an orbital table top shaker, and then centrifuged at 2,500 x g for 15 min. Methanol supernatant was transferred to a new vial, evaporated to dryness under vacuum or nitrogen at room temperature, and stored at -80°C until analysis. Extraction efficiency of a CORT standard (Enzo Life Sciences, NY) was 98% (S3 Table).

#### Feather processing and extraction

Feathers were visually inspected for external urate contamination and removed with water and gentle wiping if found. Feather vane samples were analyzed in 2 cm sections and corticosterone was extracted based on Bortollotti et al. [33], which reported >90% recovery of a radioactive corticosterone spike. Specifically, each feather section was cut into < 5mm pieces with stainless steel scissors and extracted overnight in 10 mL methanol, shaking at 50°C. Vacuum filtration through 47mm glass microfiber filters was used to remove feather material from the methanol extract. After filtration the extraction vial, filter, funnel, and collection flask were rinsed with ~3 mL additional methanol, which was added to filtered extract. Extracts were then evaporated to dryness under vacuum and stored at -80°C until analysis.

### Corticosterone and glucocorticoid metabolite measurement methods

As CORT is the primary GC in avian species [34], we tested two immunoassays that were optimized to measure CORT in rodent plasma and have also been validated in various avian species: a competitive CORT enzyme-linked immunosorbent assay (ELISA) (Cat. No. ADI-900-097, Enzo Life Sciences) and a CORT I^125^ double antibody radioimmunoassay (RIA) kit (Prod. No. 07120103, MP Biomedicals). The manufacturers’ protocols were followed, with samples being re-suspended and diluted in the respective kit’s buffers and analyzed in accordance to the manufacturer’s instructions, with the exception of using half volumes of all kit reagents in the MP Biomedicals radioimmunoassay as described in Washburn et al. [29].

An ‘analytical consistency’ sample was generated by pooling aliquots of condor plasma samples together, thoroughly mixing, sub-aliquoting into vials and storing at -80°C; an aliquot of this consistency plasma sample was included in each assay to assess within and between assay reproducibility. To test RIA interassay precision across sample types, a pooled feather extract was analyzed across assays as noted for plasma above, and nine urate samples were run on two different kits to provide a percent difference measurement (% diff) for between assay reproducibility for each kit. Standard curves for each assay day were generated by fitting measurements from serially diluted CORT standards to a 4-parameter logistic curve; CORT and GCM concentrations in samples were calculated from these standard curves. Reported non-target compound cross-reactivities for the ELISA kit antibody were 28% for deoxycorticosterone, 1.7% for progesterone, and < 1% for all other steroids tested by the manufacturer (ELISA kit manual, Enzo Life Sciences). Cross-reactivities reported for RIA kit antibody were < 1% for all steroids tested, with deoxycorticosterone having the highest tendency for cross reaction at 0.34% (RIA kit manual, MP Biomedical).

### Analytical method validation

To validate the hormone measurement methods we evaluated effects of sample matrix composition, CORT spike recovery, assay reproducibility and precision for each sample type on both ELISA and RIA [13,20]. To evaluate the effect of sample matrix composition on GC measurements we i) assessed parallelism of serial dilutions of a composite sample of each sample type (plasma, urate extract, and feather extract) with the standard curve, and ii) compared the measured hormone concentrations of serially diluted samples to their expected concentrations. To calculate the expected concentrations, we began with a sample CORT or GCM concentration from the most dilute extract, and calculated the expected concentration of the rest of the dilutions in the series using the dilution factor. For spike recovery experiments, known volumes of kit CORT standard were added to the plasma (50–460 pg CORT added), urate extract (10–280 pg CORT added), or feather extracts (30–130 pg CORT added), and analyzed in triplicate with un-spiked aliquots of the same samples for comparison (S4 Table). Thus, spike recoveries reported for these sample types reflects analytical and not total procedural (i.e. extraction efficiency and analyte quantitation) accuracy. To evaluate the analytical precision of the ELISA and RIA assays, aliquots of pooled plasma, urate, and feather samples were analyzed repeatedly within a single analytical run (intra-assay precision; n = 3–8 sample replicates run in duplicate) and across multiple analytical runs (inter-assay precision; n = 2–5 sample replicates run in duplicate).

### Statistical analyses

We used relative standard deviation values to compare precision of the immunoassays tested. Spike recoveries and other mean values are presented as mean ± standard deviation. We calculated the Pearson’s R to test for correlation to compare RIA and ELISA GC measurements. We employed a paired t-test for our validation that urate GCM increased significantly within 2 hr of a handling event. For all statistical tests, a significance level of <0.05 was used for null hypothesis rejection. Data analyses were performed using JMP Pro (Version 12 SAS Institute Inc., Cary, NC).

To determine which predictor variables influenced condor plasma CORT, or urate GCM levels we used an information theoretic approach [35] and multiple regression models. Values of continuous predictor variables were scaled before regressions by dividing by 2 times the standard deviation, so the magnitude of coefficients could be directly compared [36]. Plasma CORT response variable is reported as nanograms per milliliter (ng/mL) and urate GCM response variable as nanograms per gram dry weight (ng/g dry wt). Feather CORT was excluded from this analysis due to limited sample size.

We identified covariance between predictor variables using Spearman’s ρ correlations. We then constructed and ranked *a priori* models using second order Akaike’s Information Criterion (AIC_c_) to control for small sample sizes. Candidate models for plasma CORT and urate GCM included condor age, sex, season (spring = May 1- Jun 30; fall = Aug 1- Oct 30), keel status, hydration status, and time since stressor parameters (i.e. hours since trapped from wild, minutes since pen entry by technician, minutes since handling start). First urate GCM models also included plasma CORT as a parameter. Information on keel and hydration status was not available for all individuals (S1 Table). The decision to exclude these predictors from the final set of candidate models for first urate GCM was reached based on a finding of no significance in a prior round AIC_c_ model selection with the subset of samples for which this information was available and the goal of maximizing sample size for model inference. Both model sets included global models with all variables, and an intercept only model. The models with the lowest AIC_c_ value was considered the most parsimonious [35]. We calculated differences in AIC_c_ from the top model (ΔAIC_c_) and AIC_c_ weights for the subset of models that made up 90% of AIC_c_ weights of the complete model set. These candidate models were then used to infer the influence of each parameter. For both sets of models, we summed the Akaike weights for each parameter of influence, calculated their model averaged beta coefficients to estimate the direction and strength of their effect on GC, and determined whether parameters were informative with 90% confidence intervals.

## Results and discussion

### Immunoassay comparison and validation

#### Precision, accuracy, and matrix effect on corticosterone and glucocorticoid metabolite measurements

Generally, the ELISA and RIA assays yielded similarly acceptable intra-assay precision of the plasma CORT, feather CORT, and urate GCM measurements (Table 1). The intra-assay measurement precision ranged from 1–7% relative standard deviation (RSD) for both methods for all sample types, while the inter-assay precision for plasma was 8% RSD by ELISA versus 4.1% RSD by RIA. The inter-assay precision for pooled feather extracts analyzed across five RIA assays yielded 10% RSD and nine pooled urate extracts analyzed across two RIA assays produced an average percent difference of 6.8% (range: 1.2–14%). To assess analytical accuracy for each method, plasma, urate, and feather samples were spiked with a known amount of CORT approximately equal to the inherent sample CORT or GCM concentration (based on previous analysis or average values for sample type) before analysis, and the spike recovery was determined (S4 Table). Overall, CORT spike recoveries were ~100% (± ~1–5% SD), with several exceptions (Table 1): the CORT spike in urates was consistently under recovered as measured by ELISA (87 ± 3.0%), while RIA somewhat over-measured the CORT spike in urates (109 ± 0.8%). Also, CORT spike recovery in feather samples as measured by ELISA was more variable (119 ± 23%) than in the RIA (101 ± 4.8%).

**Table 1:** Precision and accuracy of enzyme-linked immunosorbent assay (ELISA) and radioimmunoassay (RIA) measurements of condor plasma, urate extract, and feather extract. All samples were run in duplicate and the number of samples analyzed in is in parentheses.

Method	Sample Type	Intra-assay Precision, %RSD^a^	Inter-assay Precision, %RSD^b^ or average %diff^c^	% Recovery of Corticosterone Spike^d^
**ELISA**	Plasma	6.6 (3)	7.9 (3)	101 ± 5.6 (3)
	Urates	6.2 (8)	n/a	87 ± 3.0 (5)
	Feather	2.5 (4)	n/a	119 ± 23 (3)
**RIA**	Plasma	3.2 (3)	4.1 (4)	95 ± 3.6 (3)
	Urates	1.0 (3)	6.8^c^	109 ± 0.8 (3)
	Feather	3.8 (3)	10 (5)	101 ± 4.8 (4)

^**a**^Intra-assay precision values are the percent relative standard deviation (%RSD) of sample CORT/GCM measurements analyzed within a single assay run,. Hormone concentrations were normalized to ng CORT/mL plasma, ng CORT/g feather, or ng GCM/g dry weight urate before comparison.

^**b**^Inter-assay precision is reported as %RSD for samples run on three or more assay runs over weeks to months. Samples were aliquotted and stored at -80°C before kit buffer was added, then diluted in buffer and run on same day. Hormone concentrations were normalized to ng CORT/mL plasma, ng CORT/g feather, or ng GCM/g dry weight urate before comparison.

^**c**^Inter-assay precision is reported as average % difference (%diff) for samples that were run on two assay runs. The %diff value is the average %diff for 9 samples that were run on two different assay runs (range = 1.2–14%). These samples were all dissolved in assay buffer and stored at -20°C between assay runs.

^**d**^The % spike recovery (mean ± standard deviation) reflects the percent of exogenous corticosterone added to sample prior to analysis recovered in assay measurement.

**Fig 1:**
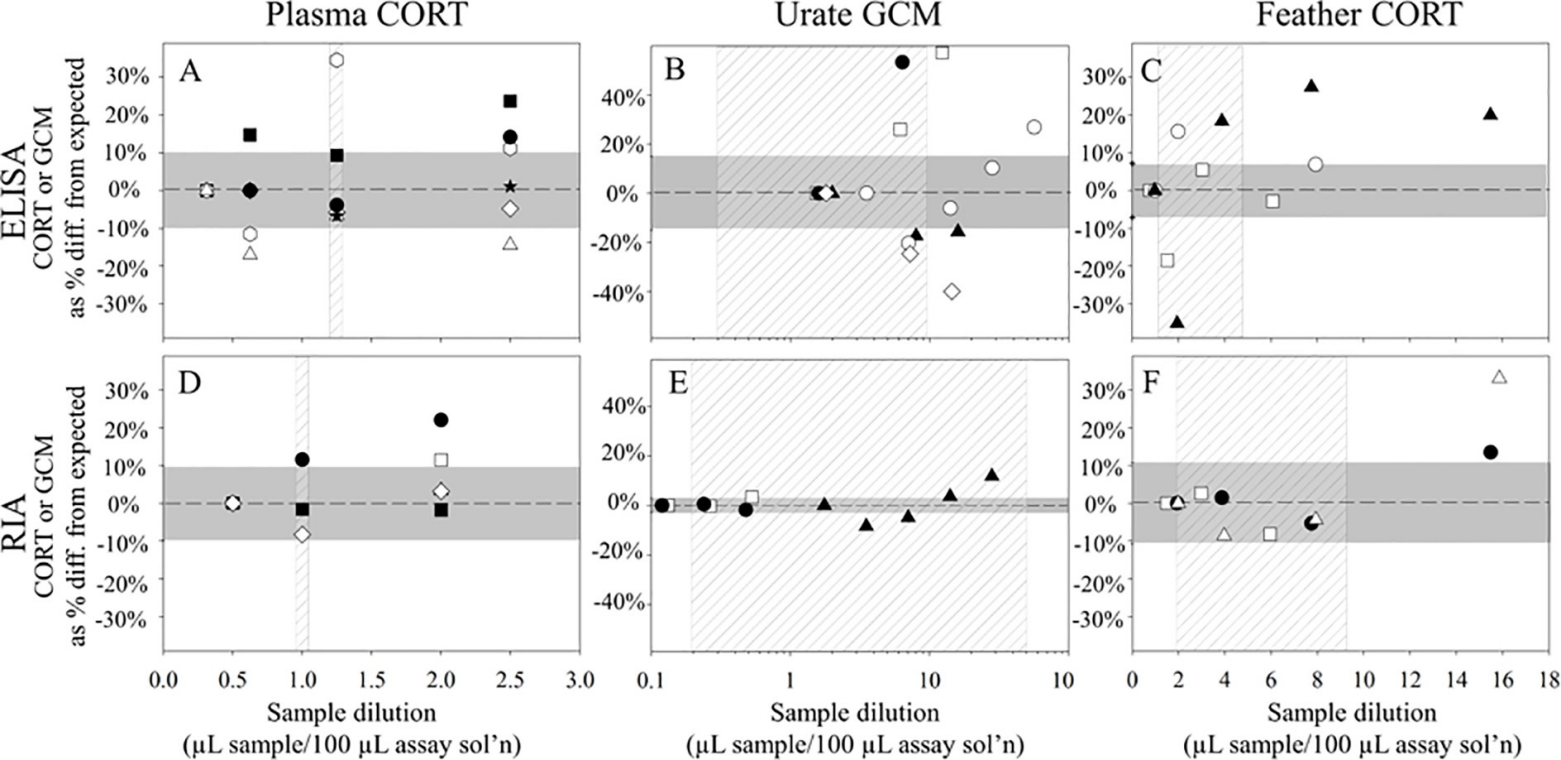
**Plasma corticosterone (CORT) (A and D), urate glucocorticoid metabolites (GCM) (B and E), and feather CORT (C and F) levels measured by radioimmunoassay (RIA) are more reproducible than enzyme-linked immunosorbent assay (ELISA) across a range of sample dilutions**. Symbols (open and filled squares, circles, triangles, and diamonds) represent a sample from an individual condor that was analyzed over a range of sample dilutions. CORT and GCM levels in diluted samples are expressed as a percent difference from expected values (horizontal dashed line at 0% diff.), based on levels measured in the most dilute sample (i.e., the lowest amount of sample in milligrams per 100 μL assay solution), and assumes sample matrix interferences are minimized in this most dilute sample. The vertical hash-marked region in each panel reflects the range of sample dilutions (x-axis) used for all samples in this study. The horizontal grey-shaded region reflects the CORT or GCM measurement uncertainty (± 2 RSD, based on intra-assay precision) for each assay and sample type; symbols within this region do not measurably differ from expected GC levels.

To test for sample matrix interference and determine the range of sample dilutions for which ELISA and RIA assays performed most reliably, parallelism tests were conducted on representative plasma, urate extract, and feather extract by serially diluting samples prior to analyses. In both ELISA and RIA, parallelism tests showed that ‘as analyzed’ concentrations of serially diluted extracts decreased linearly, parallel to the standard curve (S1 Fig). However, when we converted the ‘as analyzed’ concentrations of these serially diluted samples to ng CORT or GCM/g sample, small differences in ‘as analyzed’ concentrations compounded to create inconsistent concentrations by sample weight (Fig 1). We found measureable and unpredictable effects of sample dilution on GC concentrations by sample weight on ELISA in all three sample types, with sample concentrations diverging from expected levels in both positive and negative directions (Fig 1A–1C). In contrast, the serially diluted samples analyzed by RIA were generally within the assay measurement error (± 10%) of the expected values, particularly when sample dilutions fell within the range anticipated for typical use of the assay in this study (Fig 1D–1F). Based on these results, the CORT ELISA appears susceptible to sample matrix interferences (shown to both enhance and interfere with signal within a sample type), and thus appears less reliable for urate and feather extract analyses than the RIA.

#### Poor agreement between enzyme-linked immunoassay and radioimmunoassays for plasma corticosterone, urate glucocorticoid metabolite, and feather corticosterone measurements

**Fig 2:**
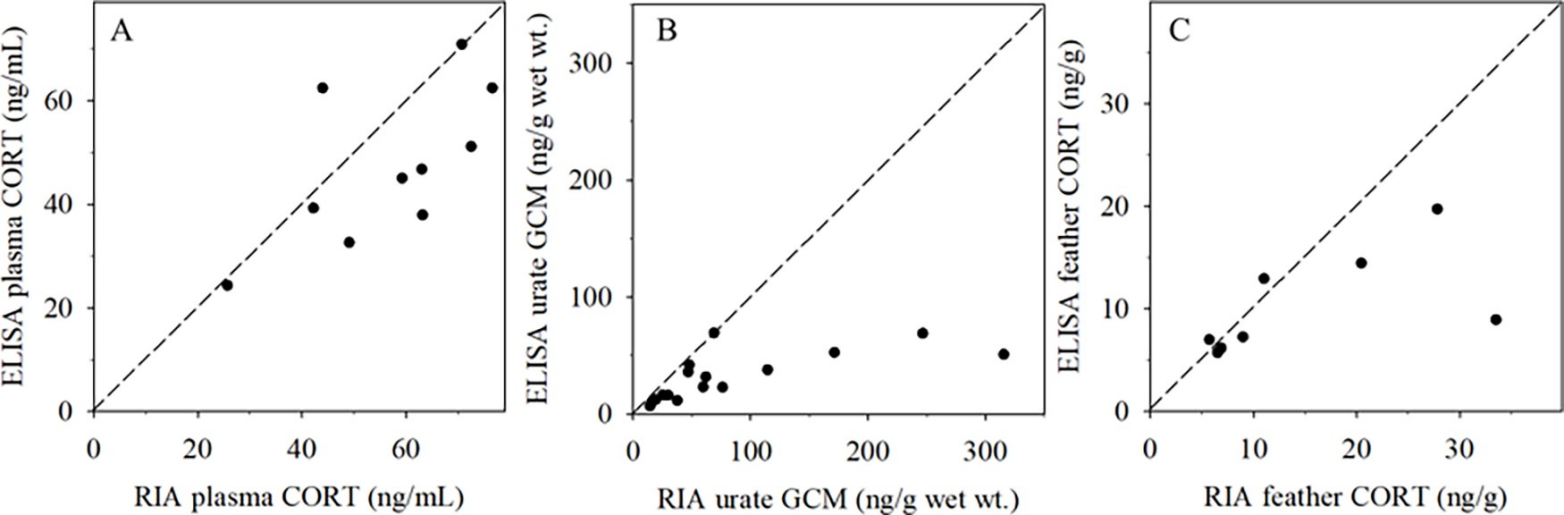
Measurements by radioimmunoassay (RIA) vs. enzyme-linked immunosorbent assay (ELISA) are different but significantly correlated for plasma corticosterone (CORT) and urate glucocorticoid metabolites (GCM). Each data point represents a condor sample measured by both RIA and ELISA. The dashed line indicates idealized agreement (y = x) between the RIA and ELISA values. In all sample types ELISA measurements trended lower than RIA measurements. (A) Plasma CORT concentrations by ELISA and RIA. (B) Urate GCM concentrations measured by RIA and ELISA, levels measured by ELISA are systematically lower by ~50%-600% compared to RIA. (C) Feather CORT concentrations measured by RIA and ELISA appear to agree only for lower CORT concentration samples (<12 ng/g).

Our results show generally poor agreement between the two methods, with the greatest disagreement typically occurring in samples with higher GC or GCM levels (Fig 2). While RIA and ELISA measurements were statistically correlated in plasma (Pearson’s R = 0.65, p = 0.04, n = 10) and urate samples (Pearson’s R = 0.70, p<0.01, n = 16), there was substantial deviation from the expected agreement for many individual samples. There was no significant correlation between GC measurements in feathers (Pearson’s R = 0.61, p = 0.11, n = 8) (Fig 2).

ELISA measurements for all sample types tended to be lower than RIA measurements. This is most apparent in urate GCM concentrations, where the median ELISA value (28 ng/g) was approximately half of the median RIA value (54 ng/g; see S5 Table for information on individual sample origins and dilutions). As the ELISA yielded urate GCM values both higher and lower than expected for the less dilute samples (Fig 1), the generally lower GCM levels measured by ELISA versus RIA (Fig 2) cannot be attributed to matrix interferences alone.

Our results suggest the ELISA kit antibody reacted with fewer metabolites, or less strongly with the metabolites compared to the RIA kit antibody. Avian plasma contains CORT in its original, secreted form, and feathers are also expected to contain predominantly CORT in secreted form along with some glucuronidated and sulfonated metabolites [33]. However, urates and feces may contain predominantly metabolized GCs with relatively little parent compound [20], and method-based differences in our GC measurements are most notable in condor urate GCM (Fig 2B). Differences in assay performance in urate extracts may be due to the different antibodies utilized in these two kits, which may have differential binding affinity with the GCMs present in condor urate samples, as has been suggested by other immunoassay comparison studies [37]. Another possibility is that matrix compounds present in condor urate and feather extracts reduce measurement accuracy by either cross-reaction with the anti-CORT antibodies, or interfering with hormone-antibody binding, as has been found with immunoassay kits and human saliva [38,39]. Previously observed inter-laboratory variation in avian plasma GC measurements indicates many potential methodological factors contribute to differences in immunoassay kit performance, yet this variation could not be explained by the use of RIA versus ELISA kits [18].

While these data do not allow us to determine outright which method is more accurate for measurement of GCs in condor samples, the superior performance of RIA across the sample dilution range suggests that RIA is more reliable than ELISA for comparing GC concentrations in condor samples.

### Handling stress challenge using radioimmunoassay to measure urate glucocorticoid metabolites and feather corticosterone

RIA’s superior reproducibility over the sample dilution ranges for urates and feather, and the generally poor agreement between the two methods, with the ELISA typically yielding much lower CORT/GCM concentrations compared to the RIA, led us to select the RIA for the handling stress challenge phase of the study. RIA has also been found to be reliable across several mammalian and avian species [14].

#### Urate glucocorticoid metabolite concentration increases in response to a stress challenge

We collected sequential urate samples for 2–6 hr following a handling event for a subset (n = 24 condors) of the condors sampled for plasma CORT (S7 Table). We found GCM concentrations were generally stable in fresh urates for at least 30 min following collection and before freezing, similar to findings by Khan et al. [40] (S2 Fig). All urate samples had detectable GCM levels (median = 710 ng/g dry wt., range = 0.74–7200 ng/g dry wt., n = 216 samples; S7 Table). Using urate sample wet weight as a proxy for volume, wet weight of urate samples in general decreased over time since handling (Spearman’s ρ = -0.19, p = 0.006, n = 216) and urate GCM concentrations on a wet weight basis were negatively correlated with sample wet weight (Spearman’s ρ = -0.61, p = 0.0001, n = 216), suggesting that GCM wet weight concentrations are influenced by volume (S3A and S3C Fig) and the excreted volume of urates may change depending on the hydration state of the bird and more (or less) fluid is reabsorbed in the lower gastrointestinal tract [41]. Conversely, urate dry weight did not significantly decrease with time (Spearman’s ρ = 0.02, p = 0.75, n = 216, S3B Fig). Additionally, urate samples of different colors (coded 1–5) had significantly different wet weight GCM concentrations (p = 0.03, n = 216, one-way ANOVA, S3D Fig), an indication of hydration state and potential fecal contamination, whereas dry weight urate GCM levels were not significantly affected by the variable of urate color (p = 0.11, n = 216, one-way ANOVA, S3E Fig). Thus, we present urate GCM levels on a dry weight basis.

**Fig 3:**
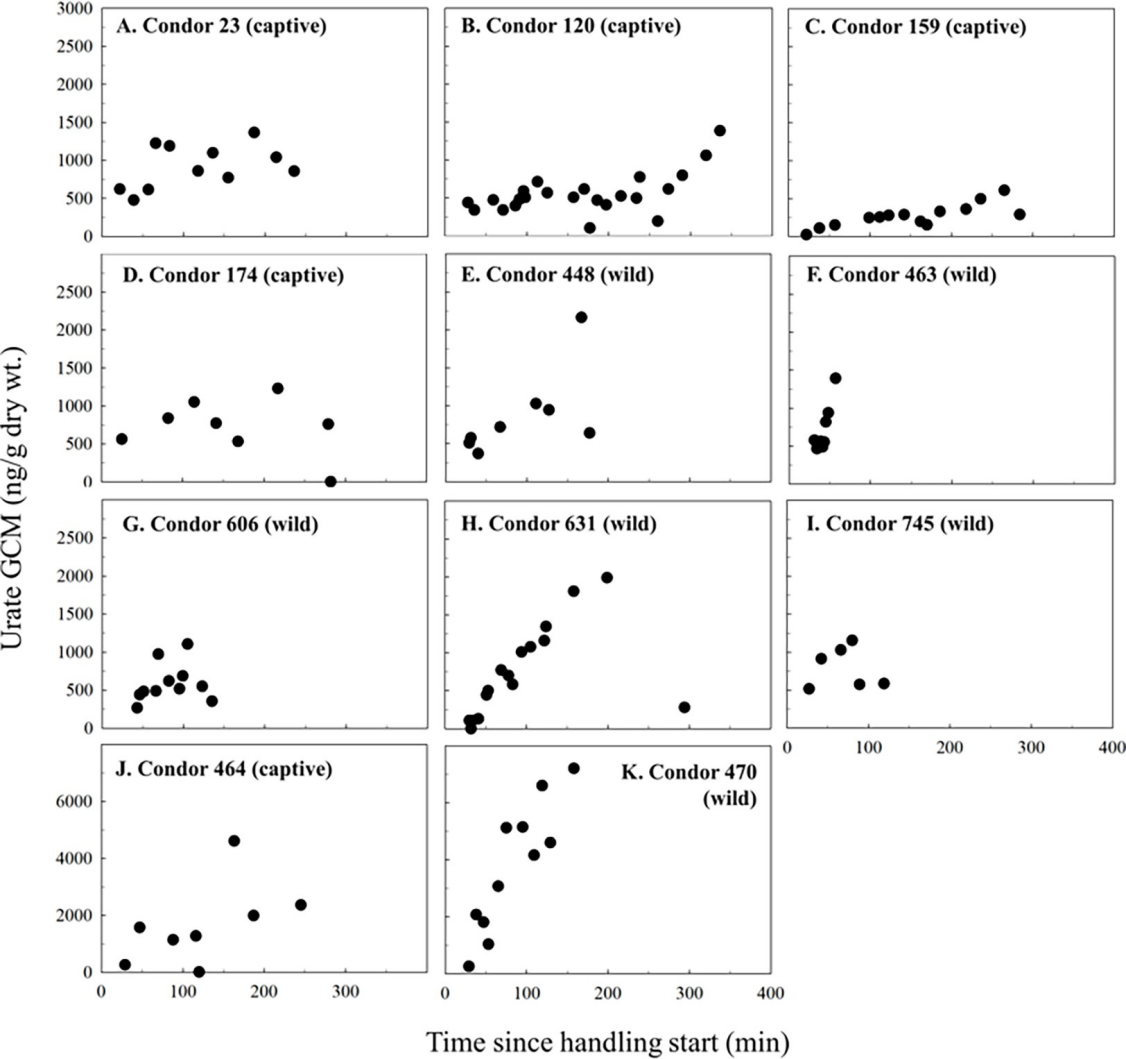
Condor urate glucocorticoid metabolite (GCM) concentrations significantly increased within 2 hours of a handling stressor. GCM concentrations (ng/g dry wt.) in condor urates collected sequentially following a physical handling and venipuncture event. Panels A-D and J are zoo-captive condors, while panels E-I and K are wild condors. X-axis shows the elapsed time since handling start; note y-axis scale difference between condor panels A-L versus J and K. See S7 Table for additional information on individual urate samples.

To measure the urate GCM response to a handling event we identified the peak GCM value within 2 hours of handling start for the 11 birds handled and kenneled for ≥2 hours. The handling stressor resulted in a significant increase in urate GCM levels over the 2 hours compared to the first urate sample collected (p = 0.032, n = 11, one-tailed paired t-test). Moreover, for most birds (n = 8), urate GCM levels remained elevated over the 3.5 hour maximum time in the kennel, with levels appearing to decline and return towards initial (i.e., first sample) GCM concentrations near the end of kenneling in some cases (n = 7, Fig 3). Hirschenhauser et al. [42] similarly reported for other avian species that after a stressor, urate GCM concentrations remained elevated for several hours. We also found that GCM levels increased ~30–40 minutes post stressor, which is comparable with Legagneux et al.’s [43] finding that GCM concentrations of snow geese (*Chen caerulescens atlantica*) feces and urates combined collected 40 min after cannon netting were significantly elevated above GCM levels in samples from undisturbed birds. Similarly, Hirschenhauser et al. [42] observed ~30 minute time lags for GCM increases in serially excreted fecal and urate samples after intravenous radiolabeled CORT injections in quail (*Coturnix japonica*) and chickens (*Gallus domesticus*). Noteworthy is that differences in elapsed time since condors were trapped from the wild or initial pen entry by a technician before the handling stressor occurred could also influence GCM levels reported in the present study, which we investigate with AIC in the following section (see *Factors influencing plasma CORT and urate GCM concentration in California condors*).

We found that the overall magnitude and pattern of change in urate GCM levels following the acute handling stressor varied widely between condors (Fig 3). In some birds, urate GCM levels increased rapidly and by several orders of magnitude (e.g., condors 631, 470, both wild), while in others the increase was slower (e.g., condors 23, 120, 159, 174, 448, 464), with the remaining three birds being somewhat intermediate between these two groups. Other avian species have also shown significant inter-individual variation in the magnitude of GCM responses to a common stressor, as measured in feces of harlequin ducks (*Histrionicus histrionicus*) [44] and greater sage grouse (*Centrocercus urophasianus*) [45]. While the underlying basis for these individual differences in the pattern/magnitude increase in urate GCM levels is not clear, it is clear that the sequential collection of urates reflects the acute stress of physical handling experienced by these condors and thus may serve as a means to monitor changes in circulating CORT levels in response to a stressor.

#### Feather corticosterone concentrations vary over time of feather growth

To determine if feather CORT concentrations vary over the period of feather growth (months) and respond to handling stressors in wild condors, we measured CORT extracted from sections of five flight feathers (four primaries, and one retrix) from five individual condors (S2 Table). For one condor (#631), 21 separate ~2 cm sections along entire length of the primary feather were analyzed. For the other four condors 3–5 ~2 cm feather sections per feather were analyzed. All 37 feather sections analyzed had measurable CORT levels (normalized to feather weight, median = 11 ng/g, range = 4.2–69 ng/g dry wt.; normalized to feather section length along the rachis, median = 18 pg/mm, range = 6.3–68 pg/mm, S2 Table), and were within the general range of feather CORT levels reported for other avian species (e.g. ~2–50 pg/mm, Bortolotti et al. [46]; ~4–370 ng/g Koren et al. [47]). To compare feather CORT levels in discrete feather sections over the period of feather growth, we normalized feather CORT levels to feather section length (mm along the rachis), since Bortolotti et al. [33,46] and Romero and Fairhurst [12] provide well-supported cases for normalizing CORT levels to feather section length along the rachis when sections of feathers are analyzed separately. We found that the mass of 2 cm feather sections of California condor primary feathers varied over the length of the feather, and hence the duration of feather growth, due to the feather’s tapered shape (S4 Fig). Feather section CORT concentrations differed depending on whether CORT levels were normalized to feather section mass or length (along the rachis), but we did not observe the decreasing trend in ng/g feather CORT over feather growth reported by Bortolotti et al. [46] (S5 Fig).

**Fig 4:**
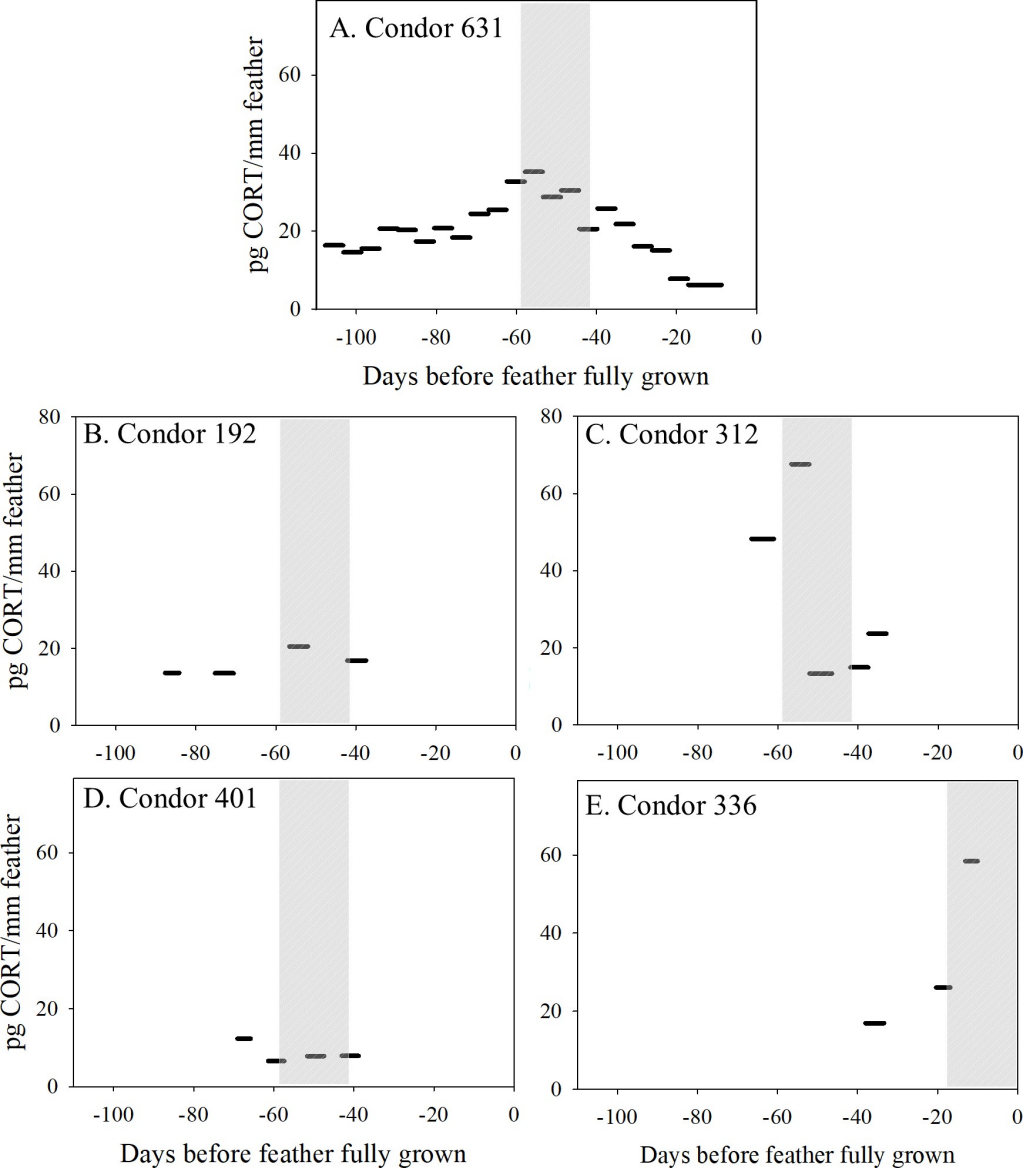
Feather section corticosterone (CORT) concentrations vary over the period of feather growth in free-flying California condors. CORT concentrations measured in sections of flight feather (2 cm lengths along rachis axis) collected from five individual wild condors. (A) Contiguous 2 cm sections from condor 631’s #6 primary feather show changes in CORT concentrations over the time of feather growth. (B-D) Non-contiguous #3 primary feather sections from condors 192, 312, and 401 show changes in feather CORT concentration over time of feather growth and variation between birds. (E) Retrix (tail) feather CORT levels from condor 336, who died of lead poisoning while receiving clinical treatment (feather growth day 0). The estimated duration (days) of feather section growth is represented for each section by the width of each section line, determined using a primary feather growth rate of 4.4 ± 0.28 mm/day in California condors [28] (See S2 Table for details). Total CORT per feather section (pg) is normalized to feather section length (mm along rachis axis) to represent integrated plasma CORT levels over time of feather section growth [33,46]. Grey shaded area indicates timing of the estimated 18 day period within which the condor was trapped, held captive in a flight pen, and handled.

Feather section CORT levels (pg CORT/mm feather) varied up to 6-fold within a feather over the ~3 month period of feather growth (Fig 4A–4E). For condor 631 for who the entire primary flight feather was analyzed, there is a clear pattern of increasing then decreasing feather CORT levels over the nearly 4 month period of feather growth (Fig 4; assumes a feather growth rate of 4.4 mm/d [28]). The other feathers also showed different patterns of CORT levels, with some (e.g., condor 192, 401) being relatively invariant over the ~30–50 day period of feather growth, while others (condors 312, 336) varied more markedly between sections (Fig 4). Analyzed condor feathers were all primaries between position 3 and 6 with the exception of a retrix (tail) feather from condor 336 (S2 Table). Prior work has illustrated that primary feathers have a fairly constant growth rate of 4.4 ± 0.28 mm/day (n = 12 feathers) (Finkelstein et al 2010). However, minor differences in true growth rates between feathers could result in added variation in feather CORT levels not accounted for here. Indeed, many factors may influence CORT deposition into growing feathers besides growth rate, including CORT release into the circulation, feather shape, the bird’s age, breeding status, and environmental stressors [12].

Because California condors are closely monitored and growing feathers often identified and marked when birds are in-hand during routine trap-ups, we were able to estimate the approximate timing of these acute stressor trapping and handling events over the period of feather growth. Feather section CORT levels increased coincident with the estimated timing of the trapping and handling event (condors 631, 192, 336; Fig 4) for three of the five condors, while for the remaining two condors the pre-handling feather CORT levels were similar to levels in feather sections collected after the trapping and handling stressor (condors 312, 401; Fig 4). Thus, while three cases suggest a possible association between trapping/handling stress and increased feather CORT levels, more cases are needed to demonstrate that stress associated with condor capture and handling produces elevated CORT deposition in condor feathers. Noteworthy is that feather CORT levels have been shown by others to reflect stress-induced increases in CORT over feather growth [33,48]. Relatively short-term changes (over 10–14 days) in feather CORT during environmental enrichment tests were observable in one study in starlings [48]. Due to the expected CORT elevation for as much as several hours after HPA axis activation in birds (e.g.[44,49]) and the greater amount of material available in California condor feathers, we rationalized that it may be possible to observe a change in CORT due to handling in this species. The markedly different patterns in feather CORT levels in these five condors may reflect that the relationship between stress-induced increases in plasma CORT and feather CORT levels varies depending on the nature of the stressor, and that the stress response induced from a ~30 min handling event may be too transient for some condors to be captured in a 2 cm feather section representing ~4–5 days of feather growth and CORT incorporation.

**Fig 5:**
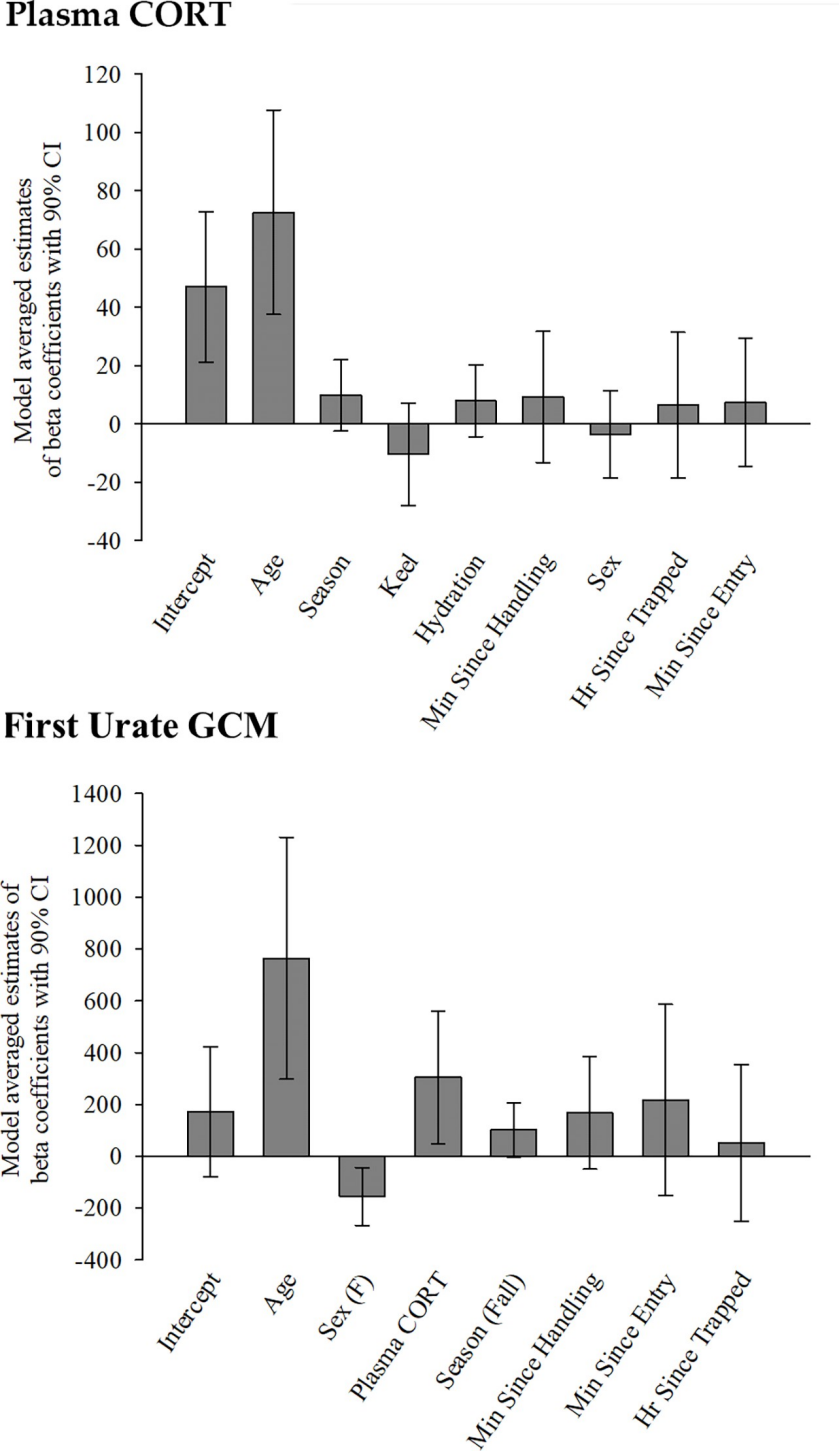
**Relative influence of predictor parameters on (A) plasma corticosterone (CORT) values, and (B) urate glucocorticoid metabolite (GCM) values in wild California condors.** Model averaged estimate of beta coefficients for all top model parameters with error bars depicting 90% confidence intervals. Parameters with confidence intervals including zero do not have sufficient support for predicting the response variable [54].

California condors are regularly lead poisoned [21], and lead exposure has been associated with altered GC stress response in birds and mammals [50,51]. Here, we measured feather CORT levels from condors for whom lead poisoning status at the time of feather collection was known. For Condors 192, 312, and 401 (Fig 4B–4D), there was no evidence of elevated lead exposure at the time of feather collection (blood leads <10 ug/dL), and general body condition was normal (S1 Table). Thus, variation in feather CORT levels in these three birds is unlikely to have been influenced by lead poisoning. In contrast, Condor 336’s feather grew during a known lead poisoning event that ultimately led to the bird’s death. Finkelstein et al. [28] estimated that condor 336 was lead exposed approximately 75 days before the growing feather was collected (i.e., around feather growth day -75, Fig 4E), and that the estimated peak blood lead level of ~1100 μg/dL occurred ~45 days before the feather was collected (i.e., at ~day -45, Fig 4E), weeks before the bird was found moribund, captured, and transported to the clinic for treatment where it died soon thereafter [28]. While only a limited number of feather sections were available for CORT analyses, they clearly show a marked increase in CORT levels that coincides with the onset of acute morbidity, capture, and clinical treatment for lead poisoning (Fig 5E). Collectively, these findings suggest that CORT feather levels hold promise as a biomarker for physiological status and/or handling-induced stress in condors, as has been established experimentally for red legged partridges (*Alectoris rufa*) [33].

### Factors influencing plasma corticosterone and urate glucocorticoid metabolite concentrations in California condors

#### Plasma corticosterone

To investigate the effects of trapping and handling stress-related factors (e.g., time since start of handling) and non-handling related factors (e.g., age, sex, body condition, season) on plasma CORT levels, we constructed and ranked *a priori* multiple linear regression models for their likelihood of predicting total (bound and unbound) plasma CORT levels. Within-species variation in CORT responses to stress is known to be influenced by many factors, including but not limited to sex, age, breeding status, season, and time of day [10]. The total plasma CORT levels measured here does not account for differing levels of corticosteroid-binding globulin (CBG) in plasma samples, but is a metric of the CORT reservoir in circulation and quantifies total biological impact of stressors [52]. All 52 plasma samples analyzed from 41 individual condors had measurable CORT concentrations (range: 1–189 ng/mL, median: 70 ng/mL; S6 Table). For individuals with multiple plasma samples collected (n = 8), one plasma sample per bird was randomly selected to be included in all statistical comparisons including the linear regression models for AIC ranking. There was a significant difference between plasma CORT values measured in captive (55 ± 31 ng/mL, n = 11 samples) compared to wild (85 ± 43 ng/mL, n = 30 samples) condors (p = 0.02, n = 41, two-tailed t-test, S5 Fig). The reason for this difference is not clear, but may be due in part to different life histories and trapping procedures for captive vs. wild condors; wild condors are lured into and trapped in the flight pen, then captured and handled for blood draw, etc., whereas captive condors are already fully acclimated to their flight pen and are simply captured and handled for their routine health checks. Given the difference between wild and captive condor plasma CORT levels, we excluded captive condors from our model selection process presented below.

In light of studies in avian species showing that plasma CORT levels begin to elevate above pre-stress baseline within 2–3 minutes of a handling stressor [23,53], the protocols for capturing the endangered condor from the wild make it difficult, if not impossible, to collect a plasma sample that represents a true physiological baseline for circulating CORT levels. In the present study, condor blood collection occurred 3–18 minutes since handling start (median = 6 min), and 10–200 min after initial flight pen entry by a technician (median = 61 min), and 1–10 days since the birds were trapped in the flight pen from the wild (median = 45 hr, range = 19–223 hr) (S6 Table). We tested how plasma CORT levels varied in response to several time points in the trapping/handling process. Since multiple wild condors are typically trapped and held in a flight pen at once, birds captured and handled later in the day experienced a greater elapsed time since the initial (for the day) pen entry by a technician, as well as a greater number of technician pen entries before being captured and handled compared to birds that were captured/handled earlier in the day. Thus, as expected, time since initial pen entry by a technician was highly correlated with the time of day of plasma collection (Spearman’s ρ = 0.63, p<0.001, n = 30), so elapsed time since initial pen entry was included in the model, but not time of day of plasma collection. The effect of elapsed time since initial pen entry might thus be confounded by a diurnal effect of time of day on plasma CORT levels.

The top two models (≤1 ΔAIC units) explaining variance in plasma CORT levels included the variables age and season (Table 2). These models of age and age + season were 2.03-times more likely than the next best model that included keel status in explaining variance in plasma CORT and explained 32–37% of the variance in plasma CORT levels (Table 2). Lower ranked models that included age plus other variables (e.g., keel status, hydration status, minutes since handling start, minutes since initial pen entry, hours since trapped in the flight pen, and sex) performed notably poorer although some models that incorporated more predictor variables (K = 5–6) explained 1–4% more variance in plasma CORT than the top models (Table 2). Notably, only age appeared to be clearly influential for plasma CORT levels, based on the 90% confidence intervals (CI) for beta coefficients for assessing variable importance in AIC model selection discussed in Arnold (2010) [54]; here, only the beta coefficients and CI’s for age did not overlap zero, while the others did (Fig 5A; S8 Table). Consistent with this, we ranked variable weights to assess relative importance in explaining variance in plasma CORT levels [35], and found models containing age had a sum AIC weight of 1.00, followed by season (0.34). The least influential variables were keel status (0.24), hydration status (0.21), minutes since handling start (0.17), sex (0.14), hours since trapped in the flight pen (0.12), and minutes since pen entry (0.11) (S8 Table). The model-averaged beta coefficient for the significant predictor variables of age is positive, indicating that plasma CORT levels increase with condor age (Fig 5A). We observed no significant influence of sex, season, keel status, or hydrations status on condor plasma GC (Fig 5A).

**Table 2:** Ranking of candidate multiple linear regression models describing variation in plasma corticosterone (CORT) concentrations and glucocorticoid metabolite (GCM) concentration of first collected urates in California condors. Within sample type, the subset of models accounting for 90% of AIC weight and the null model (intercept) are shown.

Model Structure	n	K^a^	-2 Log L	AIC_c_^b^	ΔAIC_c_^c^	w_i_^d^	Evidence ratio^e^	R^2^ ^f^
**Plasma CORT**								
age	27	3	270.179	277.222	0	0.15	1.00	0.32
age + season	27	4	267.951	277.769	0.547	0.11	1.31	0.37
age + keel	27	4	268.817	278.635	1.413	0.07	2.03	0.35
age + hydration	27	4	268.866	278.684	1.462	0.07	2.08	0.35
age + min since handling	27	4	269.752	279.57	2.348	0.05	3.23	0.33
age + min since entry	27	4	269.953	279.771	2.549	0.04	3.58	0.33
age + sex	27	4	269.987	279.805	2.583	0.04	3.64	0.32
season + hydration + age	27	5	266.988	279.845	2.623	0.04	3.71	0.40
age + hr since trapped	27	4	270.034	279.852	2.630	0.04	3.72	0.32
age + min since entry + season	27	5	267.289	280.146	2.924	0.03	4.31	0.39
age + min since handling + season	27	5	267.333	280.19	2.968	0.03	4.41	0.39
age + keel + hydration	27	5	267.527	280.384	3.162	0.03	4.86	0.38
age + season + keel	27	5	267.581	280.438	3.216	0.03	4.99	0.38
age + sex + season + keel	27	6	264.238	280.438	3.216	0.03	4.99	0.38
age + hr since trapped + season	27	5	267.861	280.718	3.496	0.03	5.74	0.38
age + sex + season	27	5	267.891	280.748	3.526	0.03	5.83	0.38
age + min since handling + keel	27	5	268.094	280.951	3.729	0.02	6.45	0.37
age + hr since trapped + hydration	27	5	268.288	281.145	3.923	0.02	7.11	0.37
age + min since handling + hydration	27	5	268.302	281.159	3.937	0.02	7.16	0.37
age + hr since trapped + keel	27	5	268.416	281.272	4.050	0.02	7.58	0.36
age + sex + hydration	27	5	268.451	281.308	4.086	0.02	7.71	0.36
age + min since entry + keel	27	5	268.478	281.335	4.113	0.02	7.82	0.36
age + sex + keel	27	5	268.566	281.423	4.201	0.02	8.17	0.36
age + min since handling + sex	27	5	269.485	282.342	5.120	0.01	12.94	0.34
age + min since entry + min since handling	27	5	269.559	282.416	5.194	0.01	13.42	0.34
age + season + min since handling + hydration	27	6	266.244	282.444	5.222	0.01	13.61	0.41
age + hr since trapped + min since handling	27	5	269.690	282.547	5.325	0.01	14.33	0.33
intercept	27	2	280.589	285.089	7.867	-	-	-
**1**^**st**^ **Urate GCM**								
age + sex	18	4	248.018	259.095	0	0.15	1.00	0.52
plasma CORT + age + sex	18	5	244.572	259.572	0.477	0.12	1.27	0.60
age + sex + season	18	5	244.769	259.769	0.674	0.11	1.40	0.60
age + min since handling + sex	18	5	245.516	260.516	1.421	0.07	2.04	0.58
plasma CORT	18	3	252.947	260.661	1.566	0.07	2.19	0.36
age + min since entry + sex	18	5	245.747	260.747	1.652	0.07	2.28	0.57
season + plasma CORT + age + sex	18	6	241.340	260.976	1.881	0.06	2.56	0.67
plasma CORT + sex	18	4	250.054	261.131	2.036	0.06	2.77	0.46
age + season	18	4	250.554	261.631	2.536	0.04	3.55	0.44
age	18	3	254.259	261.973	2.878	0.04	4.22	0.31
plasma CORT + age	18	4	250.897	261.974	2.879	0.04	4.22	0.43
plasma CORT + season	18	4	251.235	262.312	3.217	0.03	5.00	0.42
hr since trapped + plasma CORT	18	4	251.235	262.312	3.217	0.03	5.00	0.37
plasma CORT + season + age	18	5	247.316	262.316	3.221	0.03	5.01	0.53
age + min since entry	18	4	251.621	262.698	3.603	0.03	6.06	0.41
age + hr since trapped + sex	18	5	248.013	263.013	3.918	0.02	7.09	0.52
age + min since handling	18	4	252.333	263.410	4.315	0.02	8.65	0.38
min since handling + plasma CORT	18	4	252.354	263.431	4.336	0.02	8.74	0.38
intercept	18	2	261.056	265.856	6.761	-	-	-

^a^Number of estimated parameters in the model including intercept and variance.

^b^Second-order Akaike’s information criterion (AIC), optimized for small sample size.

^c^Difference in AIC_c_ value from that of most parsimonious model (i.e. model with lowest AIC_c_).

^d^Likelihood of the model relative to other models in the candidate set.

^e^Weight of evidence that the top model is better than another model, given the candidate set.

^f^ Percent of variation in plasma CORT concentration (ng/mL) explained by model.

The finding of a positive influence of age on plasma CORT in wild condors in our models was somewhat surprising given that age has been previously been shown to have no effect on plasma CORT [55] or negatively correlate with feather CORT [56] in other avian species. Thus, we investigated whether age was associated with plasma CORT levels in the captive condors (n = 11) not included in the above models and found that plasma CORT was not associated with age for the captive birds (Spearman’s ρ = -0.25, p = 0.45, S7B Fig). The contrasting effect of age on plasma CORT in the wild vs. captive condors may be due in part to the different sample size of the two groups (i.e., n = 27 vs. 11), but also suggests that in wild condors age may be a covariate for some other influential variables that increase with age. Finkelstein et al. [21] reported that condors are frequently lead poisoned over their time in the wild and we found a strong correlation between a bird’s age and their time in the wild (Spearman’s ρ = 0.94, p < .001, n = 27, S7C Fig) as expected for both wild fledged and released captive bred condors (23 of the wild condors were captive-bred). Thus, the influence of condor age on plasma CORT levels may be a surrogate of another influential variable present in the wild, such as lead exposure or other stressors. Finally, our finding that none of the stressor-related variables assessed here (i.e., minutes since handling start, minutes since initial pen entry, and hours since trapped in the flight pen) were significant predictors of plasma CORT levels was also somewhat surprising. However, rather than suggesting that these stressor events were not affecting plasma CORT levels, we think it is more likely that condor plasma CORT levels, consistent with other avian species [23,53], were already elevated to some degree by the time of blood collection (typically occurring ≥6 min after the start of physical capture/handling). Thus, we conclude that our plasma collection method cannot be used to assess baseline CORT levels, or what might more accurately be defined as ‘captive baseline’ plasma CORT levels in California condors. Our findings also highlight the need for an alternative means to assess circulating CORT levels in condors in the absence of human-induced stress, such as urates and feathers.

#### Urate glucocorticoid metabolites

As with plasma CORT above, we investigated which factors influenced urate GCM levels, by constructing a set of possible multiple linear regression models to predict GCM concentration of the first urate sample collected after the start of handling. We chose the first urate sample as our response variable since these samples were likely the least impacted by handling stressors and were most likely to reflect a ‘captive baseline’ value for comparison between individuals. We included in the models the variable plasma CORT, in addition to the variables used in the models to explain plasma CORT levels above (age, sex, season, elapsed time since trapped, elapsed time since first pen entry by a technician, and elapsed time since start of handling). We did not include the body condition variables of keel and hydration status, since those variables were only of moderate to no importance in predicting plasma CORT levels, and including them further reduced the overall number of subjects available in the models. Our top models (≤1 ΔAIC units) included the parameters age, sex, plasma CORT, and season (Table 2). The majority of the 11 models with ≤3 ΔAIC units contained age or sex (nine or seven models, respectively), while five models contained plasma CORT level, three models contained season, and one model each contained elapsed time since handling start or time since pen entry (Table 2, S9 Table). Only the variables age, sex, and plasma CORT appeared to be clearly influential for GCM levels in the first urate sample, based on the 90% confidence intervals (CI) for beta coefficients not overlapping zero (Fig 5B; S9 Table). Consistent with this, models containing the variables age, sex and plasma CORT had combined AIC weights of 0.79, 0.66, and 0.45, respectively (S9 Table) and thus were the most important with respect to explaining variance in the first urate GCM levels. In contrast, season and all three trapping/handling stressor variables (i.e., time since trapped, initial pen entry, or handling start) had notably lower combined AIC weights and did not significantly influence first urate GCM levels (Fig 5B; S9 Table). Beta coefficients show that age and plasma CORT levels were positively associated with first urate GCM levels, while sex was negatively associated, with females having lower GCM levels (Fig 5B).

Age and sex were the most supported predictor variables, accounting for 52% of the variation in first urate GCM levels, while the model containing the variables age, sex, plasma CORT and season, and explains 67% (i.e., R^2^ of 0.67) of the variance in first urate GCM levels (Table 2). None of the condors in our dataset were actively breeding or caring for young during sampling, so we expect that the sex and marginal seasonal differences (Fall ≥ Spring) we observed were due to inherent seasonal and sex-based variation in baseline GC or stress induced GC that have been well documented in other vertebrate species [57]. A positive influence of plasma CORT on the first urate CGM levels indicates that circulating CORT is related to first urate GCM measurements. Since we expect that first urate GCM measurements reflect previous levels of circulating CORT, this relationship suggests that birds that start with higher GC levels, elevate to higher GC levels, as has been documented in great tits (*Parus major*) [58]. We interpret the fact that none of the three trapping/handling stressor variables measurably predicted first urate GCM levels as evidence that the first urate sample was not measurably impacted by these stressors. The lack of association between first urate GCM levels with handling stressors suggests that the first urate sample, collected <45 min since start of handling, but typically 25–73 min since initial pen entry and 19–141 hours since trapped is not unduly influenced by these stressor variables and thus may serve as an indicator of baseline GCM levels when compared across individuals trapped from the wild.

## Conclusion

Collectively our findings highlight the need for careful validation when selecting an immunoassay method for hormone detection and measurement in a previously unstudied sample type or species, as well as caution when comparing immunoassay results across methods. We suggest that non-invasively collected urates and feathers hold promise for assessing condor responses to acute and chronic environmental and human-induced stressors and the MP Biomedicals ^125^I CORT RIA kit is appropriate for comparing hormones across sample types in the California condor.

Since endangered status of wild species can preclude the use of pharmacological challenges (i.e. ACTH) sometimes used to characterize the GC response, we suggest performing a biological method of validation for peripheral samples such as urate GCM and feather GC measurements using handling and restraint as an acute stressor. Using the RIA, we found that urate GCM and feather section CORT levels in condors generally increased following the acute stressor event of capture and handling for biannual health checks. Despite the challenges of collecting physiological GC baseline measurements for large wild species, our results show that meaningful comparisons of GC release over time can be made between individual wild condors using peripheral samples collected during handling events. Whether this finding applies to wild-captured individuals from other free-ranging species must be similarly validated.

Future studies should aim to explain more of the variability in California condor GC measurements, and the fitness outcomes of elevation or suppression of circulating GCs in this and other critically endangered species.

## Supporting information

S1 Fig Parallelism tests for corticosterone measurement in California condor plasma, urate extract, and feather extract. Corticosterone standards from kit are shown as filled black circles (●) and open circles (○) represent serially diluted samples. (A-C) Standards and samples run on ELISA kit. (D-F) Standards and samples run on RIA kit. Sample type (plasma, urate extract, or feather extract) is indicated by header above each column of plots (Panels A and D show serially diluted plasma, B and E show serially diluted urate extract, C and F show serially diluted feather extract).
(PDF)

S2 Fig Urate GCM concentrations appear stable up to 30 minutes. Four urate samples from three California condors (two samples from one individual) were homogenized via shaking in the field and aliquoted into 2–3 vials. One vial was immediately placed on dry ice after collection (< 8 min since defecation), whereas the remaining vials were placed on dry ice at ~15 and ~30 minutes after collection. Error bars show 6.2% RSD (intra-assay precision for urates by ELISA) and illustrate no measureable change in urate GCM concentration within 30 minutes, except for in the 692 #4 where a measurable difference was detected between ASAP vs. 15 min to freezing (37% RSD).
(PDF)

S3 Fig **Rationale for using dry weight concentrations for urate GCM**: (A) We observed a decrease in wet weight (g) of sample over time since handling (Spearman’s ρ = -0.19, p = 0.006, n = 216), (B) but not for sample dry weights (g) (Spearman’s ρ = 0.02, p = 0.75, n = 216). (C) Wet weight GCM concentration was also negatively correlated with sample wet weight (Spearman’s ρ = -0.61, p< 0.0001, n = 216). (D) Urate samples of different colors (coded 1–5, ranging from 1 = white/clear, 3 = yellow, 5 = green) had significantly different wet weight GCM concentrations (p = 0.03, n = 216, one-way ANOVA), an indicator of hydration and potential fecal contamination, whereas dry weight urate GCM were not significantly affected by this variable (p = 0.11, n = 216, one-way ANOVA). Taken together, this evidence suggests that wet wt. GCM concentrations in urates are more sensitive to hydration states of the individual than dry wt. GCM concentrations. We therefor used ng/g dry wt. for GCM concentrations for our condor urate results.
(PDF)

S4 Fig Mass of 2 cm feather vane sections vary along the length of a condor primary feather. Primary feathers have a tapered shape that causes the amount of feather grown for a given time period to vary over feather growth (Bortolotti et al. 2009).
(PDF)

S5 Fig Feather CORT concentrations per gram of feather show similar results to CORT concentrations normalized to feather section length (Fig 4 main text, see also S2 Table). (PDF)

S6 Fig Plasma GC (RIACort) values measured in captive (55 ± 31 ng/mL, n = 11 samples) vs. wild (85 ± 43 ng/mL, n = 30 samples) condors are significantly different (p = 0.02, two-tailed t test). (PDF)

S7 Fig Correlates of plasma CORT. (A)Age is correlated with plasma CORT (RIACort) in wild condors (Spearman’s ρ = 0.48, p = 0.01, n = 27). (B) Age is not correlated with Plasma CORT (RIACort) in captive condors (Spearman’s ρ = -0.25, p = 0.45, n = 11). (C) For wild condors, age may be a covariate for other influential variables that increases with time in wild (FreeFlyDays) (Spearman’s ρ = 0.94, p < .001, n = 27).
(PDF)

S1 Table California condors sampled. a. F = female, M = male
b. Age in years
c. captive = chick hatched and fledged in captivity; wild = chick hatched and fledged in the wild
d. Condor’s status at time of sample collection: wild = free-flying in the wild population; captive = long-term captive in zoo facility; captive* = in the captive population during sample collection but slated for release to the wild population
e. Sample collection locations; LAZ = Los Angeles Zoo and Botanical Park, CA; VWS = Ventana Wildlife Society trapping site in Big Sur, CA; PNP = trapping site in Pinnacles National Park, CA
f. Trap date for wild condors. Not applicable (NA) for captive condors which live in flight pens continuously before handling events.
g. Weight at sample collection, not available for all condors.
h. Keel rating is an indicator of body condition measured by palpating keel and pectoral muscle. Condors are scored 1–5 where 1 = emaciated, severely atrophied pectoral muscles in relationship to keel bone, 2 = keel protrudes slightly beyond pectoral muscles, 3 = average, pectoral muscles approximately even with keel bone, 4 = pectoral muscles robust and extend beyond keel bone, 5 = obese, pectoral muscles unusually robust and extend well beyond keel bone. Not scored in all captive birds, and not provided for feather collections. To minimize technician-related bias, we coded these categorical observations as binary for statistical analysis (Keel status: 0 = breast concave to keel, 1 = breast muscle even or convex to keel.)
i. Hydration status: 0 = dehydrated, 1 = well hydrated based on leg skin elasticity after pinching.
(PDF)

S2 Table Details for California condor feather sections. a. Feather position code: R/L = right/left, P = primary, # = primary feather position, “retrix” = tail feather of unknown position.
b. Distance from start of feather section to skin (incorporates exposed calamus length)
c. Section length along rachis axis of feather
d. Days of feather growth/section. Calculated based on feather section length using 0.0441 cm/day growth rate for California condor primary feathers [28]
e. These two time points bracket the predicted duration of feather growth (days for which the feather material in this section was perfused during formation in follicle). Based on feather growth calculations from columns A and C.
(PDF)

S3 Table CORT extraction recovery California condor urates. Urate samples were pooled and aliquoted before spiking with corticosterone. All samples were lyophilized and extracted with either 80% MeOH or 95% EtOH and GCM concentration of re-suspended extracts were measured by ELISA. Based on results 80% MeOH was used as urate extraction method.
a. Unspiked aliquots were extracted by each solvent and their GCM concentration averaged to calculate spike recovery in spiked samples (80% MeOH: n = 4 unspiked samples, 95% EtOH: n = 3 samples).
b. Included to provide insight into endogenous: spiked hormone ratio as run on ELISA.
c. % spike recovery calculated by first subtracting total endogenous GCM from total GCM plus CORT measured in spiked aliquots of pooled urates, then comparing the difference to the known weight of hormone in added spike (ng) (80% MeOH: n = 4 spiked aliquots, 95% EtOH: n = 3 spiked aliquots). Total ng endogenous GCM was calculated for spiked samples by multiplying mean endogenous GCM concentration of unspiked samples by aliquot wet weight (g).
(PDF)

S4 Table Analytical corticosterone (CORT) spike recovery data. a. For feather and urates: mg sample dry/100mL assay buffer
b. For urates: mg sample wet/100mL assay buffer; for plasma: μL sample /100mL assay buffer
c. Plasma CORT, feather CORT, or urate GCM concentration as run (pg/tube for RIA, ng/mL assay buffer for ELISA) d. Exogenous corticosterone spike in ng as run.
(PDF)

S5 Table Samples used in RIA vs. ELISA method comparison. a. For feather and urates: mg sample dry/100mL assay buffer
b. For urates: mg sample wet/100mL assay buffer; for plasma: μL sample /100mL assay buffer
c. Plasma CORT, feather CORT, or urate GCM concentration as run (ng hormone/mL assay buffer)
d. Plasma CORT or urate GCM concentration in wet sample (ng hormone/mL plasma or ng hormone/g urates wet wt.)
e. Feather CORT, or urate GCM concentration in dry sample (ng hormone/g dry wt. for urates and feather)
f. Total ng CORT or GCM in sample.
(PDF)

S6 Table Collection and CORT data for plasma samples. a. Time of sample collection as hours since bird was trapped from the wild. Condors are caught and moved into flight pen using a double door trap operated from a blind, and therefor do not see a human until the flight pen entry by technicians on handling days.
b. Time of sample collection as minutes since initial flight pen entry by technicians. This precedes handling start.
c. Time of sample collection as minutes since handling start. Handling start was recorded when condor was trapped in hoop net.
(PDF)

S7 Table Collection and GCM data for urate samples. a. Time of sample collection as hours since bird was trapped from the wild. Condors are caught and moved into flight pen using a double door trap operated from a blind, and therefor do not see a human until the flight pen entry by technicians on handling days.
b. Time of sample collection as minutes since initial flight pen entry by technicians. This precedes handling start.
c. Time of sample collection as minutes since handling start. Handling start was recorded when condor was trapped in hoop net.
(PDF)

S8 Table Multiple linear regression model averaged parameter estimates for plasma CORT levels. a. Number of competitive models (listed in Table 2) including the parameter.
b. Summed Akaike weights for all models with parameter.
c. Weighted average beta coefficient.
d. Model averaged standard error.
e. 90% confidence interval for parameter estimate.
(PDF)

S9 Table Multiple linear regression model averaged parameter estimates for 1^st^ urate GCM. a. Number of competitive models (listed in Table 2) including the parameter.
b. Summed Akaike weights for all models with parameter.
c. Weighted average beta coefficient.
d. Model averaged standard error.
e. 90% confidence interval for parameter estimate.
(PDF)
