# Supplementary material for: Glucocorticoid measurement in plasma, urates, and feathers from California condors (Gymnogyps californianus) in response to a human-induced stressor
Source: PLoS One. 2018 Oct 23;13(10):e0205565. doi: 10.1371/journal.pone.0205565 (PMC6198957; doi:10.1371/journal.pone.0205565)

S1 Fig. Parallelism tests for corticosterone measurement in California condor plasma, urate extract, and feather extract. Corticosterone standards from kit are shown as filled black circles (●) and open circles (○) represent serially diluted samples. (A-C) Standards and samples run on ELISA kit. (D-F) Standards and samples run on RIA kit. Sample type (plasma, urate extract, or feather extract) is indicated by header above each column of plots (Panels A and D show serially diluted plasma, B and E show serially diluted urate extract, C and F show serially diluted feather extract).

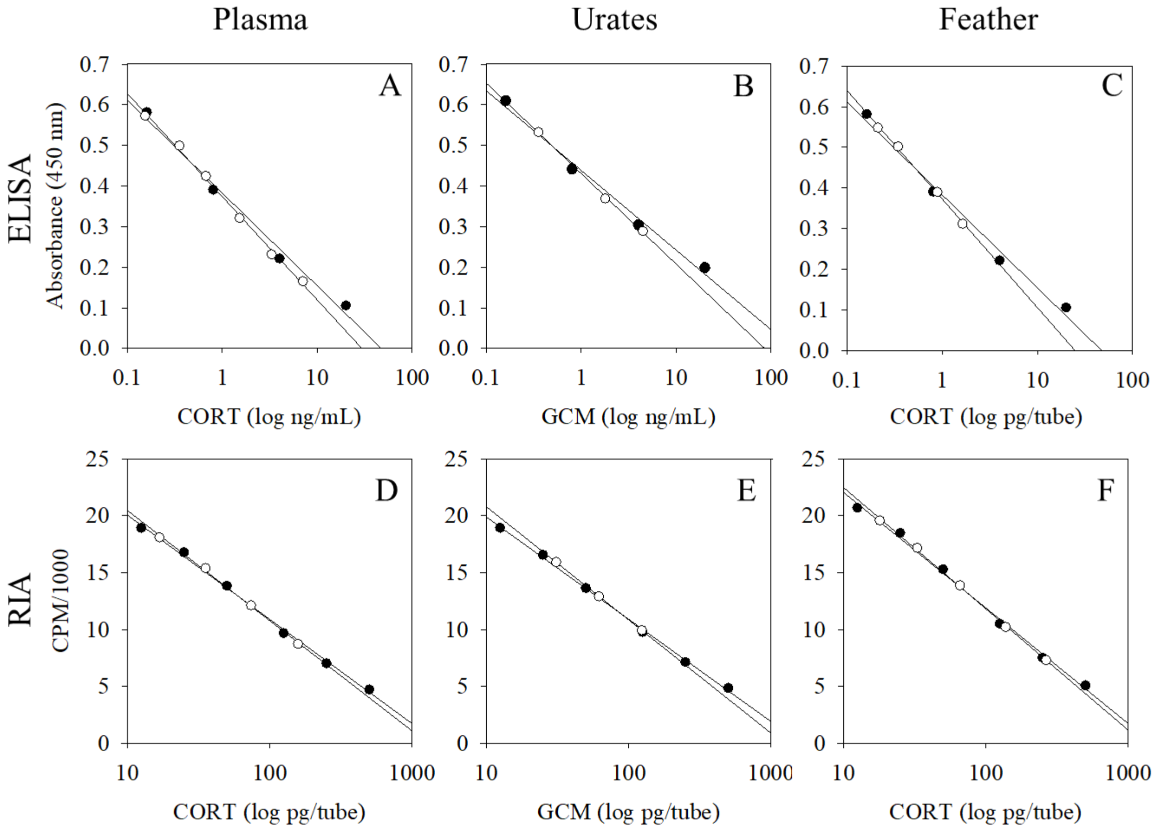

Supplement: S1 Fig — Corticosterone standards from kit are shown as filled black circles (●) and open circles (○) represent serially diluted samples. (A-C) Standards and samples run on ELISA kit. (D-F) Standards and samples run on RIA kit. Sample type (plasma, urate extract, or feather extract) is indicated by header above each column of plots (Panels A and D show serially diluted plasma, B and E show serially diluted urate extract, C and F show serially diluted feather extract). (PDF) [file pone.0205565.s001.pdf]
