# Supplementary material for: Glucocorticoid measurement in plasma, urates, and feathers from California condors (Gymnogyps californianus) in response to a human-induced stressor
Source: PLoS One. 2018 Oct 23;13(10):e0205565. doi: 10.1371/journal.pone.0205565 (PMC6198957; doi:10.1371/journal.pone.0205565)

S2 Fig. Urate GCM concentrations appear stable up to 30 minutes. Four urate samples from three California condors (two samples from one individual) were homogenized via shaking in the field and aliquoted into 2-3 vials. One vial was immediately placed on dry ice after collection (< 8 min since defecation), whereas the remaining vials were placed on dry ice at ~15 and ~30 minutes after collection. Error bars show 6.2% RSD (intra-assay precision for urates by ELISA) and illustrate no measureable change in urate GCM concentration within 30 minutes, except for in the 692 #4 where a measurable difference was detected between ASAP vs. 15 min to freezing (37% RSD).

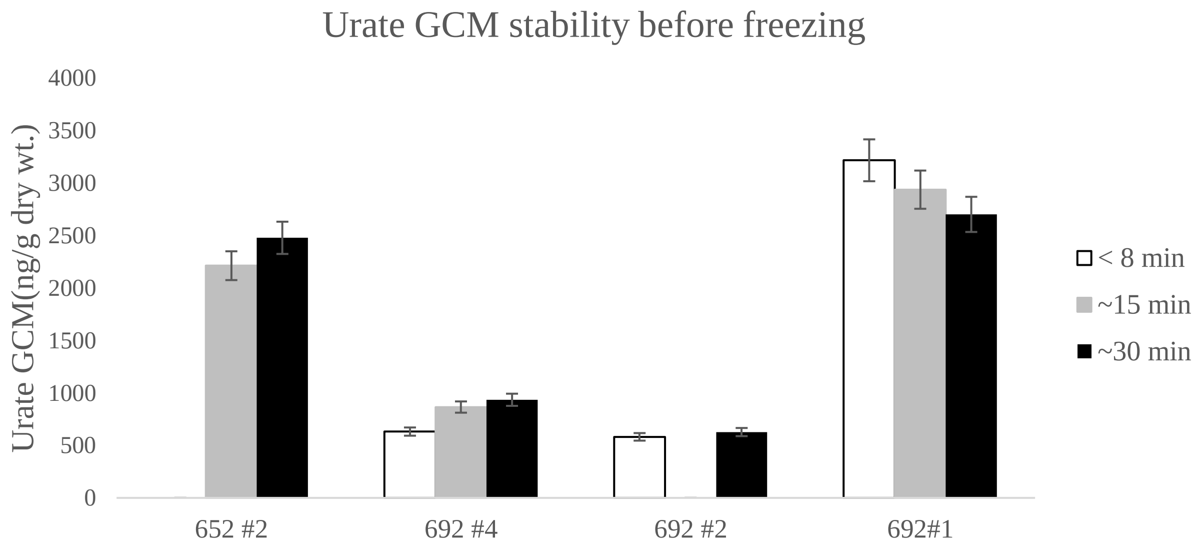

Supplement: S2 Fig — Four urate samples from three California condors (two samples from one individual) were homogenized via shaking in the field and aliquoted into 2–3 vials. One vial was immediately placed on dry ice after collection (< 8 min since defecation), whereas the remaining vials were placed on dry ice at ~15 and ~30 minutes after collection. Error bars show 6.2% RSD (intra-assay precision for urates by ELISA) and illustrate no measureable change in urate GCM concentration within 30 minutes, except for in the 692 #4 where a measurable difference was detected between ASAP vs. 15 min to freezing (37% RSD). (PDF) [file pone.0205565.s002.pdf]
