# Supplementary material for: Glucocorticoid measurement in plasma, urates, and feathers from California condors (Gymnogyps californianus) in response to a human-induced stressor
Source: PLoS One. 2018 Oct 23;13(10):e0205565. doi: 10.1371/journal.pone.0205565 (PMC6198957; doi:10.1371/journal.pone.0205565)

S3 Fig. Rationale for using dry weight concentrations for urate GCM: (A) We observed a decrease in wet weight (g) of sample over time since handling (Spearman's ρ = -0.19, p= 0.006, n= 216), (B) but not for sample dry weights (g) (Spearman's ρ = 0.02, p = 0.75, n= 216). (C) Wet weight GCM concentration was also negatively correlated with sample wet weight (Spearman's ρ = -0.61, p = < 0.0001, n= 216). (D) Urate samples of different colors (coded 1-5, ranging from 1=white/clear, 3=yellow, 5=green) had significantly different wet weight GCM concentrations (p=0.03, n=216, one-way ANOVA), an indicator of hydration and potential fecal contamination, whereas dry weight urate GCM were not significantly affected by this variable (p=0.11, n=216, one-way ANOVA). Taken together, this evidence suggests that wet wt. GCM concentrations in urates are more sensitive to hydration states of the individual than dry wt. GCM concentrations. We therefor use ng/g dry wt. for GCM concentrations for our condor urate results.

Panel A.

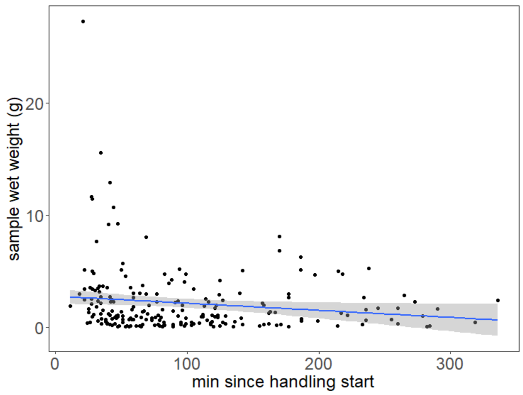

Panel B.

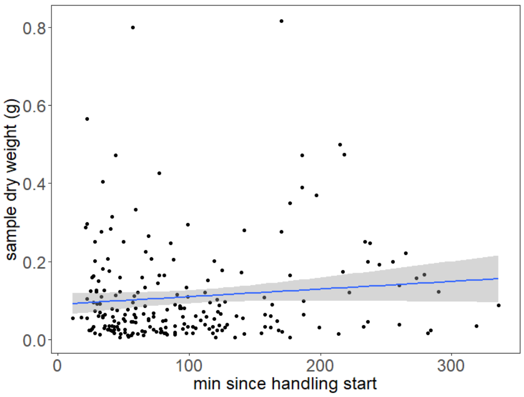

Panel C.

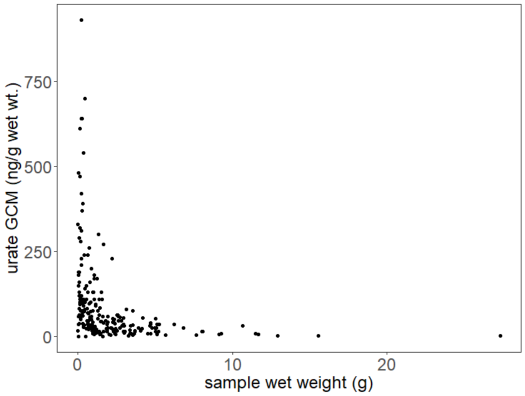

Panel D.

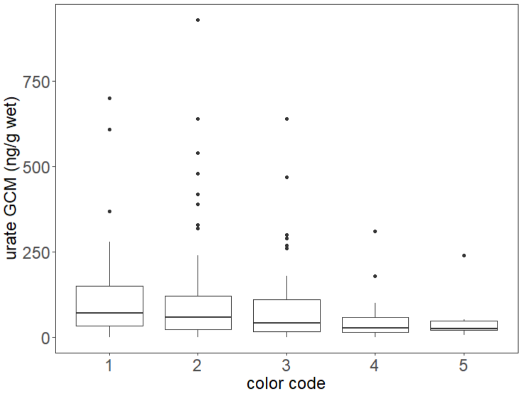

Panel E.

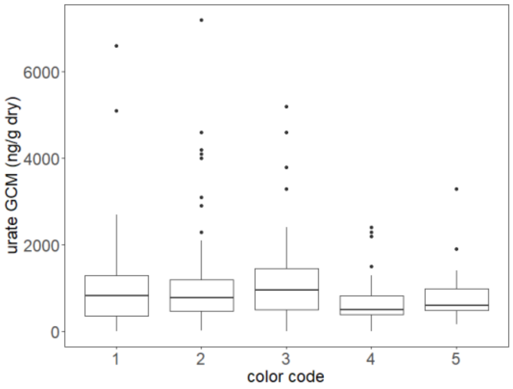

Supplement: S3 Fig — Rationale for using dry weight concentrations for urate GCM: (A) We observed a decrease in wet weight (g) of sample over time since handling (Spearman’s ρ = -0.19, p = 0.006, n = 216), (B) but not for sample dry weights (g) (Spearman’s ρ = 0.02, p = 0.75, n = 216). (C) Wet weight GCM concentration was also negatively correlated with sample wet weight (Spearman’s ρ = -0.61, p< 0.0001, n = 216). (D) Urate samples of different colors (coded 1–5, ranging from 1 = white/clear, 3 = yellow, 5 = green) had significantly different wet weight GCM concentrations (p = 0.03, n = 216, one-way ANOVA), an indicator of hydration and potential fecal contamination, whereas dry weight urate GCM were not significantly affected by this variable (p = 0.11, n = 216, one-way ANOVA). Taken together, this evidence suggests that wet wt. GCM concentrations in urates are more sensitive to hydration states of the individual than dry wt. GCM concentrations. We therefor used ng/g dry wt. for GCM concentrations for our condor urate results. (PDF) [file pone.0205565.s003.pdf]
