# Supplementary material for: Glucocorticoid measurement in plasma, urates, and feathers from California condors (Gymnogyps californianus) in response to a human-induced stressor
Source: PLoS One. 2018 Oct 23;13(10):e0205565. doi: 10.1371/journal.pone.0205565 (PMC6198957; doi:10.1371/journal.pone.0205565)
Supplement: S4 Fig — Primary feathers have a tapered shape that causes the amount of feather grown for a given time period to vary over feather growth (Bortolotti et al. 2009). (PDF) [file pone.0205565.s004.pdf]

S4 Fig. Mass of 2 cm feather vane sections vary along the length of a condor primary feather. Primary feathers have a tapered shape that causes the amount of feather grown for a given time period to vary over feather growth (Bortolotti et al. 2009).

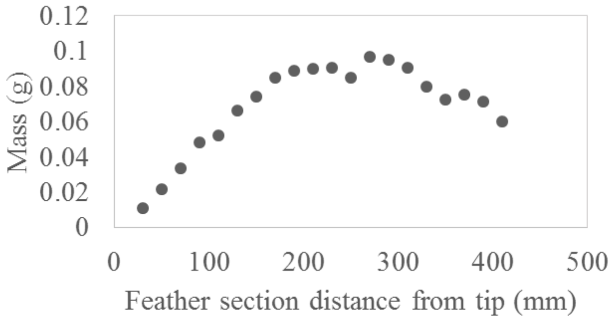

Bortolotti, G. R., T. Marchant, J. Blas, and S. Cabezas (2009). Tracking stress: localisation, deposition and stability of corticosterone in feathers. The Journal of Experimental Biology 212:1477–82. doi: 10.1242/jeb.022152
