# Supplementary material for: Glucocorticoid measurement in plasma, urates, and feathers from California condors (Gymnogyps californianus) in response to a human-induced stressor
Source: PLoS One. 2018 Oct 23;13(10):e0205565. doi: 10.1371/journal.pone.0205565 (PMC6198957; doi:10.1371/journal.pone.0205565)

S5 Fig. Feather CORT concentrations per gram of feather show similar results to CORT concentrations normalized to feather section length (Figure 6 main text, see also SI Table 2).

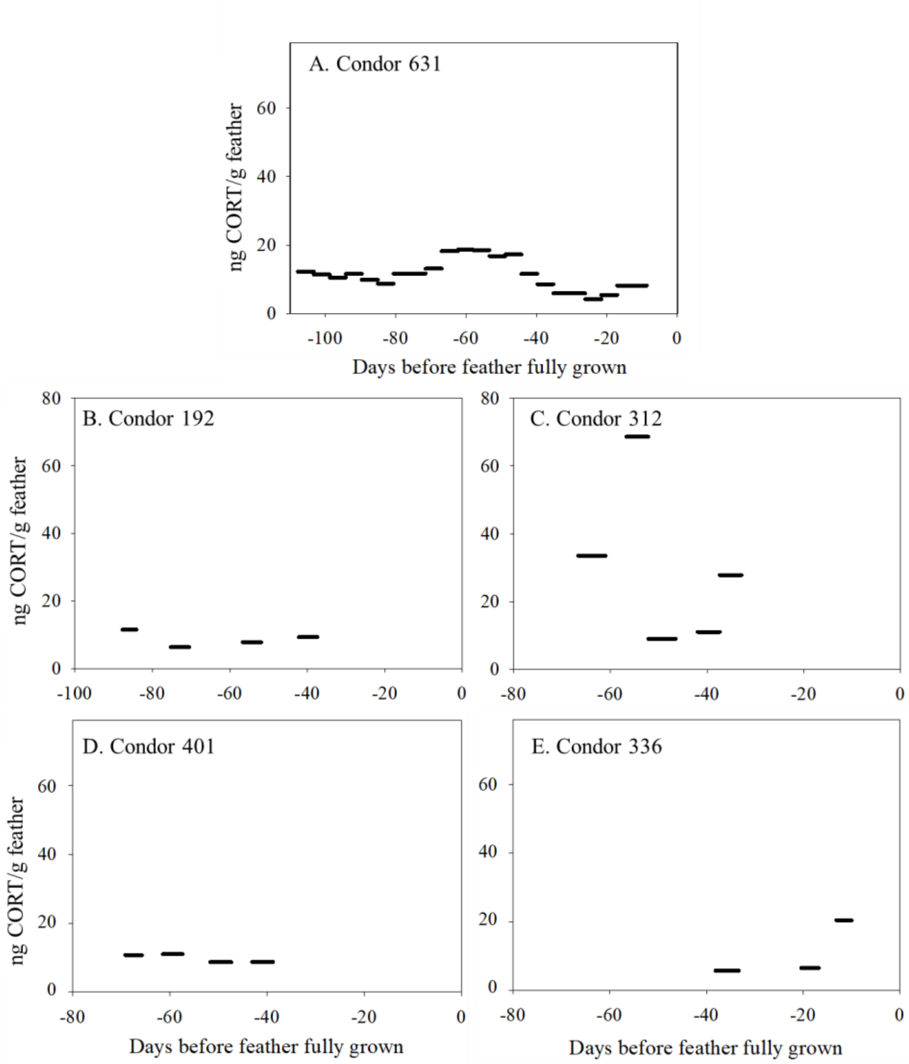

Supplement: S5 Fig — (PDF) [file pone.0205565.s005.pdf]
