# Supplementary material for: Glucocorticoid measurement in plasma, urates, and feathers from California condors (Gymnogyps californianus) in response to a human-induced stressor
Source: PLoS One. 2018 Oct 23;13(10):e0205565. doi: 10.1371/journal.pone.0205565 (PMC6198957; doi:10.1371/journal.pone.0205565)

S6 Fig. Plasma GC (RIACort) values measured in captive (55 ± 31 ng/mL, n=11 samples) vs. wild (85 ± 43 ng/mL, n=30 samples) condors are significantly different(p=0.02, two-tailed t test).

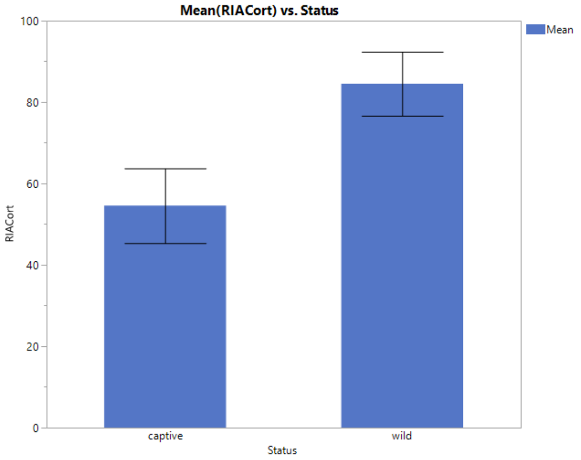

Supplement: S6 Fig — (PDF) [file pone.0205565.s006.pdf]
