# Supplementary material for: Glucocorticoid measurement in plasma, urates, and feathers from California condors (Gymnogyps californianus) in response to a human-induced stressor
Source: PLoS One. 2018 Oct 23;13(10):e0205565. doi: 10.1371/journal.pone.0205565 (PMC6198957; doi:10.1371/journal.pone.0205565)

S7 Fig. (A)Age is correlated with plasma CORT (RIACort) in wild condors (Spearman's ρ=0.48, p=0.01, n=27). (B) Age is not correlated with Plasma CORT (RIACort) in captive condors (Spearman's ρ=-0.25, p=0.45, n=11). (C) For wild condors, age may be a covariate for other influential variables that increases with time in wild (FreeFlyDays) (Spearman's ρ=0.94, p<.001, n=27).

Panel A.

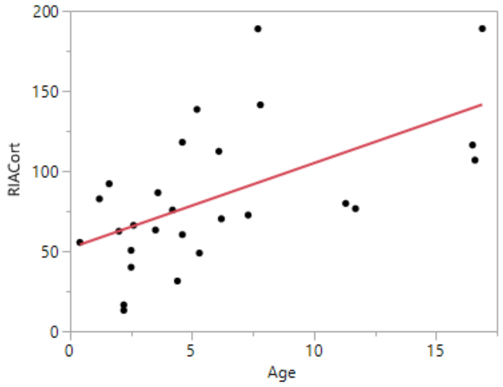

Panel B.

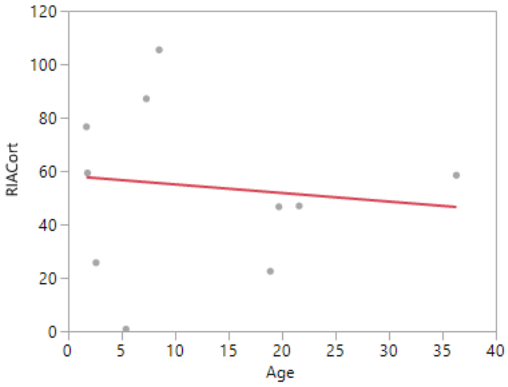

Panel C.

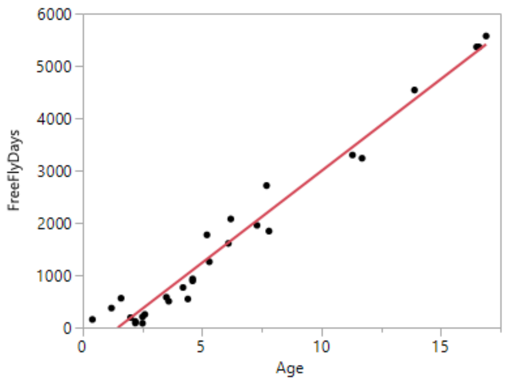

Supplement: S7 Fig — (A)Age is correlated with plasma CORT (RIACort) in wild condors (Spearman’s ρ = 0.48, p = 0.01, n = 27). (B) Age is not correlated with Plasma CORT (RIACort) in captive condors (Spearman’s ρ = -0.25, p = 0.45, n = 11). (C) For wild condors, age may be a covariate for other influential variables that increases with time in wild (FreeFlyDays) (Spearman’s ρ = 0.94, p < .001, n = 27). (PDF) [file pone.0205565.s007.pdf]
