# Supplementary material for: Glucocorticoid measurement in plasma, urates, and feathers from California condors (Gymnogyps californianus) in response to a human-induced stressor
Source: PLoS One. 2018 Oct 23;13(10):e0205565. doi: 10.1371/journal.pone.0205565 (PMC6198957; doi:10.1371/journal.pone.0205565)
Supplement: S1 Table — a. F = female, M = male b. Age in years c. captive = chick hatched and fledged in captivity; wild = chick hatched and fledged in the wild d. Condor’s status at time of sample collection: wild = free-flying in the wild population; captive = long-term captive in zoo facility; captive* = in the captive population during sample collection but slated for release to the wild population e. Sample collection locations; LAZ = Los Angeles Zoo and Botanical Park, CA; VWS = Ventana Wildlife Society trapping site in Big Sur, CA; PNP = trapping site in Pinnacles National Park, CA f. Trap date for wild condors. Not applicable (NA) for captive condors which live in flight pens continuously before handling events. g. Weight at sample collection, not available for all condors. h. Keel rating is an indicator of body condition measured by palpating keel and pectoral muscle. Condors are scored 1–5 where 1 = emaciated, severely atrophied pectoral muscles in relationship to keel bone, 2 = keel protrudes slightly beyond pectoral muscles, 3 = average, pectoral muscles approximately even with keel bone, 4 = pectoral muscles robust and extend beyond keel bone, 5 = obese, pectoral muscles unusually robust and extend well beyond keel bone. Not scored in all captive birds, and not provided for feather collections. To minimize technician-related bias, we coded these categorical observations as binary for statistical analysis (Keel status: 0 = breast concave to keel, 1 = breast muscle even or convex to keel.) i. Hydration status: 0 = dehydrated, 1 = well hydrated based on leg skin elasticity after pinching. (PDF) [file pone.0205565.s008.pdf]

S1 Table. California condors sampled

Condor ID	Sex[a]	Age[b]	Hatch origin[c]	Status at sampling[d]	Location[e]	Trap Date[f]	Sample collection date	Weight at capture (lbs)[g]	Keel rating[h]	Hydration status[i]
23	M	36	wild	captive	LAZ	NA	6/14/2016	17.7	NA	NA
120	M	21	captive	captive	LAZ	NA	6/14/2016	18.6	NA	NA
159	F	19	captive	captive	LAZ	NA	6/14/2016	18.3	NA	NA
174	F	18	captive	captive	SBZ	NA	7/28/2016	NA	NA	NA
192	F	18	captive	wild	LAZ	NA	6/26/2010	NA	NA	NA
199	M	18	captive	wild	VWS	6/2/2015	6/3/2015	NA	3	0
204	M	12	captive	wild	VWS	6/2/2015	6/3/2015	NA	3	0
209	M	16	captive	wild	VWS	10/28/2015	10/29/2015	22	3	0
236	F	15	captive	wild	PNP	10/8/2015	10/14/2015	17.5	3	0
312	F	6	captive	wild	PNP	NA	5/27/2009	NA	NA	NA
336	F	4	captive	wild	LAZ	NA	9/7/2008	NA	NA	NA
340	M	11	captive	wild	PNP	10/12/2015	10/14/2015	17.8	3	0
351	M	11	captive	wild	PNP	6/9/2015	6/10/2015	17.8	2	0
401	M	3	captive	wild	PNP	NA	5/27/2009	NA	NA	NA
411	M	8	captive	wild	PNP	10/26/2014	10/29/2014	19	3	0
448	M	7	captive	wild	PNP	6/15/2014	6/16/2014	17.9	3	1
463	M	7	captive	wild	PNP	10/26/2015	10/28/2015	19	3	0
464	F	8	captive	captive	SBZ	7/26/2016	7/28/2016	NA	NA	NA
470	M	7	wild	wild	VWS	10/28/2015	10/29/2015	NA	3	0
477	M	7	wild	wild	VWS	5/27/2015	5/28/2015	NA	2	NA
538	F	6	wild	wild	PNP	5/23/2015	5/27/2015	16.4	2	1
544	F	7	captive	captive	SBZ	NA	7/28/2016	NA	NA	NA
547	F	5	captive	wild	VWS	6/2/2015	6/3/2015	NA	3	0
564	M	5	captive	wild	VWS	5/19/2015	6/3/2015	NA	2	1
567	M	5	wild	wild	VWS	5/19/2015	5/28/2015	NA	3	1
583	F	4	captive	wild	PNP	5/5/2015	5/6/2015	16.5	2	0
597	F	4	captive	wild	PNP	10/2/2015	10/7/2015	19.5	4	1
603	F	5	wild	captive	SBZ	NA	7/28/2016	NA	NA	NA
606	M	4	captive	wild	VWS	10/18/2015	10/21/2015	18.4	3	0
615	M	3	captive	wild	PNP	5/30/2014	6/4/2014	19.6	2	1
626	F	3	captive	wild	PNP	10/27/2014	10/29/2014	19.8	4	0
631	M	2	captive	captive*	LAZ	NA	1/7/2014	NA	4	NA
631	M	4	captive	wild	PNP	10/8/2015	10/14/2015	20	4	1
631	M	4	captive	wild	PNP	NA	11/12/2015	NA	NA	NA
646	F	1	wild	captive*	LAZ	NA	1/7/2014	NA	NA	NA
650	M	1	captive	captive*	LAZ	NA	1/7/2014	NA	NA	NA
650	M	3	captive	wild	PNP	10/8/2015	10/14/2015	18.3	3	0
652	M	1	captive	captive*	LAZ	NA	1/7/2014	NA	NA	NA
652	M	2	captive	wild	VWS	10/22/2014	10/23/2014	NA	3	2
684	F	2	captive	wild	PNP	6/21/2015	6/23/2015	20.6	3	0

Condor ID	Sex[a]	Age[b]	Hatch origin[c]	Status at sampling[d]	Location[e]	Trap Date[f]	Sample collection date	Weight at capture (lbs)[g]	Keel rating[h]	Hydration status[i]
684	F	2	captive	wild	PNP	10/12/2015	10/14/2015	18	3	1
687	F	2	captive	wild	PNP	10/5/2015	10/7/2015	20	4	1
688	M	2	captive	wild	VWS	6/2/2015	6/3/2015	20.6	3	0
692	M	2	captive	wild	PNP	6/9/2015	6/10/2015	NA	3	1
700	M	2	captive	wild	PNP	10/2/2015	10/7/2015	18.6	5	1
704	M	2	captive	wild	PNP	10/20/2015	10/21/2015	21.3	4	1
729	M	1	wild	wild	PNP	10/9/2015	10/14/2015	20.6	3	0
745	M	1	wild	wild	VWS	5/27/2015	5/28/2015	NA	3	1
769	F	1	wild	wild	VWS	6/2/2015	6/3/2015	19.7	3	0

a. F = female, M = male
b. Age in years
c. captive = chick hatched and fledged in captivity; wild = chick hatched and fledged in the wild
d. Condor's status at time of sample collection: wild = free-flying in the wild population; captive = long-term captive in zoo facility; captive* = in the captive population during sample collection but slated for release to the wild population
e. Sample collection locations; LAZ = Los Angeles Zoo and Botanical Park, CA; VWS = Ventana Wildlife Society trapping site in Big Sur, CA; PNP = trapping site in Pinnacles National Park, CA
f. Trap date for wild condors. Not applicable (NA) for captive condors which live in flight pens continuously before handling events.
g. Weight at sample collection, not available for all condors.
h. Keel rating is an indicator of body condition measured by palpating keel and pectoral muscle. Condors are scored 1-5 where 1 = emaciated, severely atrophied pectoral muscles in relationship to keel bone, 2 = keel protrudes slightly beyond pectoral muscles, 3 = average, pectoral muscles approximately even with keel bone, 4 = pectoral muscles robust and extend beyond keel bone, 5 = obese, pectoral muscles unusually robust and extend well beyond keel bone. Not scored in all captive birds, and not provided for feather collections. To minimize technician-related bias, we coded these categorical observations as binary for statistical analysis (Keel status: 0= breast concave to keel, 1= breast muscle even or convex to keel.)
i. Hydration status: 0= dehydrated, 1=well hydrated based on leg skin elasticity after pinching.
