# Supplementary material for: Glucocorticoid measurement in plasma, urates, and feathers from California condors (Gymnogyps californianus) in response to a human-induced stressor
Source: PLoS One. 2018 Oct 23;13(10):e0205565. doi: 10.1371/journal.pone.0205565 (PMC6198957; doi:10.1371/journal.pone.0205565)
Supplement: S2 Table — a. Feather position code: R/L = right/left, P = primary, # = primary feather position, “retrix” = tail feather of unknown position. b. Distance from start of feather section to skin (incorporates exposed calamus length) c. Section length along rachis axis of feather d. Days of feather growth/section. Calculated based on feather section length using 0.0441 cm/day growth rate for California condor primary feathers [28] e. These two time points bracket the predicted duration of feather growth (days for which the feather material in this section was perfused during formation in follicle). Based on feather growth calculations from columns A and C. (PDF) [file pone.0205565.s009.pdf]

S2 Table. Details for California condor feather sections.

Condor ID	Feather position[a]	Date Coll.	Section #	Feather length from skin[b] (mm)	Mass (g)	Total CORT (ng)	CORT (ng/g)	Section length (cm)[c]	Days of feather growth[c]	pg CORT/ mm feather	Section start[d] (days before full grown)	Section extent[e] (days before full grown)
192	LP3	6/26/2010	1 proximal	51	-	-	-	3.4	7.7	-	-11.6	-19.27
192	LP3	6/26/2010	2	85	0.0286	-	-	2	4.5	-	-19.27	-23.81
192	LP3	6/26/2010	3	105	-	-	-	2.0	4.5	-	-23.81	-28.34
192	LP3	6/26/2010	4	125	-	-	-	2.0	4.5	-	-28.34	-32.88
192	LP3	6/26/2010	5	145	0.0411	-	-	2.0	4.5	-	-32.88	-37.41
192	LP3	6/26/2010	6	165	0.0379	0.35	9.3	2.1	4.8	17	-37.41	-42.18
192	LP3	6/26/2010	7	186	-	-	-	2.0	4.5	-	-42.18	-46.71
192	LP3	6/26/2010	8	206	0.0304	-	-	2.3	5.2	-	-46.71	-51.93
192	LP3	6/26/2010	9	229	0.055	0.43	7.8	2.1	4.8	20	-51.93	-56.69
192	LP3	6/26/2010	10	250	-	-	-	2.0	4.5	-	-56.69	-61.22
192	LP3	6/26/2010	11	270	0.027	-	-	2.1	4.8	-	-61.22	-65.99
192	LP3	6/26/2010	12	291	-	-	-	2.0	4.5	-	-65.99	-70.52
192	LP3	6/26/2010	13	311	0.045	0.28	6.3	2.1	4.8	14	-70.52	-75.28
192	LP3	6/26/2010	14	332	0.0308	-	-	1.9	4.3	-	-75.28	-79.59
192	LP3	6/26/2010	15	351	-	-	-	2.0	4.5	-	-79.59	-84.13
192	LP3	6/26/2010	16	371	0.0187	0.22	12	1.6	3.6	14	-84.13	-87.76
192	LP3	6/26/2010	17+18 distal	387	0.0171	-	-	3.8	8.6	-	-87.76	-96.37
312	RP3	5/27/2009	1+2 proximal	45	0.0242	-	-	4.0	9.1	-	-10.2	-19.3
312	RP3	5/27/2009	3	85	-	-	-	2.0	4.5	-	-19.3	-23.8
312	RP3	5/27/2009	4	105	-	-	-	2.0	4.5	-	-23.8	-28.3
312	RP3	5/27/2009	5	125	0.017	-	-	2.0	4.5	-	-28.3	-32.9
312	RP3	5/27/2009	6	145	0.017	0.47	28	2.0	4.5	24	-32.9	-37.4
312	RP3	5/27/2009	7	165	0.027	0.3	11	2.0	4.5	15	-37.4	-42.0
312	RP3	5/27/2009	8	185	0.0183	-	-	2.0	4.5	-	-42.0	-46.5

Condor ID	Feather position[a]	Date Coll.	Section #	Feather length from skin[b] (mm)	Mass (g)	Total CORT (ng)	CORT (ng/g)	Section length (cm)[c]	Days of feather growth[c]	pg CORT/ mm feather	Section start[d] (days before full grown)	Section extent[e] (days before full grown)
312	RP3	5/27/2009	9	205	0.037	0.33	9.0	2.5	5.7	13	-46.5	-52.2
312	RP3	5/27/2009	10	230	0.0364	1.35	69	2.0	4.5	68	-52.2	-56.7
312	RP3	5/27/2009	11	250	0.0223	-	-	1.9	4.3	-	-56.7	-61.0
312	RP3	5/27/2009	12	269	0.036	1.21	34	2.5	5.7	48	-61.0	-66.7
312	RP3	5/27/2009	13	294	-	-	-	1.0	2.3	-	-66.7	-68.9
312	RP3	5/27/2009	14	304	0.0173	-	-	1.8	4.1	-	-68.9	-73.0
312	RP3	5/27/2009	15	322	-	-	-	2.6	5.9	-	-73.0	-78.9
312	RP3	5/27/2009	16	348	-	-	-	1.1	2.5	-	-78.9	-81.4
312	RP3	4/12/2009	17 distal	359	0.057	-	-	5.9	13.4	-	-81.4	-94.8
336	retrix	9/7/2008	1+2 proximal	0	0.0419	-	-	3.0	6.8	-	0.0	-6.8
336	retrix	9/7/2008	3	30	0.0393	-	-	1.4	3.2	-	-6.8	-10.0
336	retrix	9/7/2008	4	44	0.0397	0.82	20	1.4	3.2	58	-10.0	-13.2
336	retrix	9/7/2008	5	58	0.0537	-	-	1.6	3.6	-	-13.2	-16.8
336	retrix	9/7/2008	6	74	0.062	0.42	6.5	1.6	3.6	26	-16.8	-20.4
336	retrix	9/7/2008	7	90	0.0585	-	-	1.8	4.1	-	-20.4	-24.5
336	retrix	9/7/2008	8	108	0.0481	-	-	1.8	4.1	-	-24.5	-28.6
336	retrix	9/7/2008	9	126	0.0476	-	-	2.1	4.8	-	-28.6	-33.3
336	retrix	9/7/2008	10	147	0.062	0.36	5.7	2.1	4.8	17	-33.3	-38.1
336	retrix	9/7/2008	11	168	0.0482	-	-	1.9	4.3	-	-38.1	-42.4
336	retrix	9/7/2008	12	187	0.0466	-	-	2.0	4.5	-	-42.4	-46.9
336	retrix	9/7/2008	13 distal	207	0.0647	-	-	4.0	9.1	-	-46.9	-56.0
401	RP3	5/27/2009	1+2 proximal	50	-	-	-	3.5	7.9	-	-11.3	-19.3
401	RP3	5/27/2009	3	77	0.0147	-	-	2.0	4.5	-	-17.5	-22.0
401	RP3	5/27/2009	4	94	-	-	-	2.0	4.5	-	-21.3	-25.9
401	RP3	5/27/2009	5	116	0.0163	-	-	2.0	4.5	-	-26.3	-30.8
401	RP3	5/27/2009	6	134	-	-	-	2.0	4.5	-	-30.4	-34.9

Condor ID	Feather position[a]	Date Coll.	Section #	Feather length from skin[b] (mm)	Mass (g)	Total CORT (ng)	CORT (ng/g)	Section length (cm)[c]	Days of feather growth[c]	pg CORT/ mm feather	Section start[d] (days before full grown)	Section extent[e] (days before full grown)
401	RP3	5/27/2009	7	152	0.0197	-	-	2.0	4.5	-	-34.5	-39.0
401	RP3	5/27/2009	8	171	0.0178	0.16	8.8	2.0	4.5	7.9	-38.8	-43.3
401	RP3	5/27/2009	9	190	0.0194	-	-	2.0	4.5	-	-43.1	-47.6
401	RP3	5/27/2009	10	209	0.0182	0.16	8.7	2.0	4.5	7.8	-47.4	-51.9
401	RP3	5/27/2009	11	228	0.017	-	-	2.0	4.5	-	-51.7	-56.2
401	RP3	5/27/2009	12	253	0.012	0.13	11	2.0	4.5	6.6	-57.4	-61.9
401	RP3	5/27/2009	13	271	0.0283	-	-	2.0	4.5	-	-61.5	-66.0
401	RP3	5/27/2009	14	290	0.0231	0.25	11	2.0	4.5	12	-65.8	-70.3
401	RP3	5/27/2009	15	305	0.0252	-	-	2.0	4.5	-	-69.2	-73.7
401	RP3	5/27/2009	16	324	0.023	-	-	2.0	4.5	-	-73.5	-78.0
401	RP3	5/27/2009	17+18	342	0.0298	-	-	4.0	9.1	-	-77.6	-86.6
401	RP3	5/27/2009	19 distal	380	0.0605	-	-	3.8	8.6	-	-86.2	-94.8
631	RP6	11/12/2015	1+2 proximal	38	0.0286	0.23	8.1	3.7	8.4	6.3	-8.7	-17.1
631	RP6	11/12/2015	3	75	0.029	0.16	5.4	2.0	4.5	7.8	-17.1	-21.6
631	RP6	11/12/2015	4	95	0.0725	0.30	4.2	2.0	4.5	15	-21.6	-26.2
631	RP6	11/12/2015	5	115	0.0551	0.32	5.8	2.0	4.5	16	-26.2	-30.7
631	RP6	11/12/2015	6	135	0.0741	0.44	5.9	2.0	4.5	22	-30.7	-35.2
631	RP6	11/12/2015	7	155	0.0604	0.52	8.6	2.0	4.5	26	-35.2	-39.8
631	RP6	11/12/2015	8	175	0.0355	0.41	12	2.0	4.5	21	-39.8	-44.3
631	RP6	11/12/2015	9	195	0.0353	0.61	17	2.0	4.5	30	-44.3	-48.8
631	RP6	11/12/2015	10	215	0.0343	0.58	17	2.0	4.5	29	-48.8	-53.4
631	RP6	11/12/2015	11	235	0.0381	0.70	18	2.0	4.5	35	-53.4	-57.9
631	RP6	11/12/2015	12	255	0.0352	0.65	19	2.0	4.5	33	-57.9	-62.4
631	RP6	11/12/2015	13	275	0.0281	0.51	18	2.0	4.5	26	-62.4	-67.0
631	RP6	11/12/2015	14	295	0.0374	0.49	13	2.0	4.5	24	-67.0	-71.5
631	RP6	11/12/2015	15	315	0.0315	0.37	12	2.0	4.5	18	-71.5	-76.1

Condor ID	Feather position[a]	Date Coll.	Section #	Feather length from skin[b] (mm)	Mass (g)	Total CORT (ng)	CORT (ng/g)	Section length (cm)[c]	Days of feather growth[c]	pg CORT/ mm feather	Section start[d] (days before full grown)	Section extent[e] (days before full grown)
631	RP6	11/12/2015	16	335	0.0358	0.42	12	2.0	4.5	21	-76.1	-80.6
631	RP6	11/12/2015	17	355	0.0399	0.35	8.7	2.0	4.5	17	-80.6	-85.1
631	RP6	11/12/2015	18	375	0.0412	0.41	9.9	2.0	4.5	20	-85.1	-89.7
631	RP6	11/12/2015	19	395	0.0359	0.41	12	2.0	4.5	21	-89.7	-94.2
631	RP6	11/12/2015	20	415	0.0296	0.31	10	2.0	4.5	16	-94.2	-98.7
631	RP6	11/12/2015	21	435	0.0258	0.29	11	2.0	4.5	15	-98.7	-103.3
631	RP6	11/12/2015	22 distal	455	0.0268	0.33	12	2.0	4.5	16	-103.3	-107.8

a. Feather position code: R/L = right/left, P=primary, # = primary feather position, “retrix”= tail feather of unknown position.
b. Distance from start of feather section to skin (incorporates exposed calamus length)
c. Section length along rachis axis of feather
d. Days of feather growth/section. Calculated based on feather section length using 0.0441 cm/day growth rate for California condor primary feathers (Finkelstein et al. 2010)
e. These two time points bracket the predicted duration of feather growth (days for which the feather material in this section was perfused during formation in follicle). Based on feather growth calculations from columns A and C.
