# Supplementary material for: Glucocorticoid measurement in plasma, urates, and feathers from California condors (Gymnogyps californianus) in response to a human-induced stressor
Source: PLoS One. 2018 Oct 23;13(10):e0205565. doi: 10.1371/journal.pone.0205565 (PMC6198957; doi:10.1371/journal.pone.0205565)
Supplement: S3 Table — Urate samples were pooled and aliquoted before spiking with corticosterone. All samples were lyophilized and extracted with either 80% MeOH or 95% EtOH and GCM concentration of re-suspended extracts were measured by ELISA. Based on results 80% MeOH was used as urate extraction method. a. Unspiked aliquots were extracted by each solvent and their GCM concentration averaged to calculate spike recovery in spiked samples (80% MeOH: n = 4 unspiked samples, 95% EtOH: n = 3 samples). b. Included to provide insight into endogenous: spiked hormone ratio as run on ELISA. c. % spike recovery calculated by first subtracting total endogenous GCM from total GCM plus CORT measured in spiked aliquots of pooled urates, then comparing the difference to the known weight of hormone in added spike (ng) (80% MeOH: n = 4 spiked aliquots, 95% EtOH: n = 3 spiked aliquots). Total ng endogenous GCM was calculated for spiked samples by multiplying mean endogenous GCM concentration of unspiked samples by aliquot wet weight (g). (PDF) [file pone.0205565.s010.pdf]

S3 Table. CORT extraction recovery for California condor urates. Urate samples were pooled and aliquoted before spiking with corticosterone. All samples were lyophilized and extracted with either 80% MeOH or 95% EtOH and GCM concentration of re-suspended extracts were measured by ELISA. Based on results 80% MeOH was used as urate extraction method.

Extraction Solvent	Mean endogenous GCM concentration (ng/g)[a]	sd	Mean endogenous total GCM in aliquot (ng)[b]	CORT spike (ng)	% spike recovery[c]	sd
**80% methanol**	19	7.8	5.0	9.2	98	22
**95% ethanol**	14	2.6	3.6	9.2	43	1.5

a. Unspiked aliquots were extracted by each solvent and their GCM concentration averaged to calculate spike recovery in spiked samples (80% MeOH: n=4 unspiked samples, 95% EtOH: n=3 samples).
b. Included to provide insight into endogenous: spiked hormone ratio as run on ELISA.
c. % spike recovery calculated by first subtracting total endogenous GCM from total GCM plus CORT measured in spiked aliquots of pooled urates, then comparing the difference to the known weight of hormone in added spike (ng) (80% MeOH: n=4 spiked aliquots, 95% EtOH: n=3 spiked aliquots). Total ng endogenous GCM was calculated for spiked samples by multiplying mean endogenous GCM concentration of unspiked samples by aliquot wet weight (g).
