# Supplementary material for: Glucocorticoid measurement in plasma, urates, and feathers from California condors (Gymnogyps californianus) in response to a human-induced stressor
Source: PLoS One. 2018 Oct 23;13(10):e0205565. doi: 10.1371/journal.pone.0205565 (PMC6198957; doi:10.1371/journal.pone.0205565)
Supplement: S4 Table — a. For feather and urates: mg sample dry/100mL assay buffer b. For urates: mg sample wet/100mL assay buffer; for plasma: μL sample /100mL assay buffer c. Plasma CORT, feather CORT, or urate GCM concentration as run (pg/tube for RIA, ng/mL assay buffer for ELISA) d. Exogenous corticosterone spike in ng as run. (PDF) [file pone.0205565.s011.pdf]

S4 Table. Analytical corticosterone (CORT) spike recovery data

Sample ID	Condor ID if applicable	Sample Type	Assay	Dilution factor (dry)[a]	Dilution factor (wet)[b]	CORT or GCM as run[c]	ng CORT or GCM as run	CORT spike (ng)[d]	% CORT spike recovery
Const_P	pooled	plasma	ELISA	NA	1.5	1.01	0.10	NA	NA
Const_P	pooled	plasma	ELISA	NA	1.5	0.93	0.09	NA	NA
Const_P	pooled	plasma	ELISA	NA	1.5	0.86	0.09	NA	NA
Const_P_sp	pooled	plasma	ELISA	NA	1.5	4.89	0.49	0.46	86
Const_P_sp	pooled	plasma	ELISA	NA	1.5	5.06	0.51	0.46	90
Const_P_sp	pooled	plasma	ELISA	NA	1.5	4.85	0.49	0.46	85
F_unsp	pooled	feather	ELISA	4.7	NA	0.58	0.06	NA	NA
F_unsp	pooled	feather	ELISA	4.7	NA	0.51	0.05	NA	NA
F_unsp	pooled	feather	ELISA	4.7	NA	0.62	0.06	NA	NA
F_sp	pooled	feather	ELISA	4.7	NA	2.42	0.24	0.13	139
F_sp	pooled	feather	ELISA	4.7	NA	2.24	0.22	0.13	125
F_sp	pooled	feather	ELISA	4.7	NA	1.82	0.18	0.13	94
650 #4d	650	urates	ELISA	0.2	2.0	0.83	0.08	NA	NA
650 #4ds	650	urates	ELISA	0.2	2.0	6.44	0.64	0.28	198
652 #4d	652	urates	ELISA	0.2	1.8	1.24	0.12	NA	NA
652 #4ds	652	urates	ELISA	0.2	1.8	6.44	0.64	0.28	183
448 #7ds	448	urates	ELISA	0.1	1.5	0.35	0.04	NA	NA
448 #7ds	448	urates	ELISA	0.1	1.5	3.35	0.33	0.28	106
UP1_19	pooled	urates	ELISA	3.1	37.7	1.11	0.11	NA	NA
UP1_20	pooled	urates	ELISA	3.2	36.4	0.64	0.06	NA	NA
UP1_21	pooled	urates	ELISA	3.1	37.1	0.78	0.08	NA	NA
UP1_22	pooled	urates	ELISA	3.2	38.8	0.73	0.07	NA	NA
UP1_23	pooled	urates	ELISA	1.4	18.2	0.84	0.08	0.04	73
UP1_24	pooled	urates	ELISA	1.4	16.7	0.77	0.08	0.04	67
UP1_25	pooled	urates	ELISA	1.5	16.1	0.80	0.08	0.04	73
UP1_26	pooled	urates	ELISA	1.7	17.7	1.10	0.11	0.04	117
UP1_27	pooled	urates	ELISA	1.3	16.8	1.01	0.10	0.04	105
Feath336pooled_1	336	feather	RIA	33.0	NA	30.96	0.03	NA	NA

Sample ID	Condor ID if applicable	Sample Type	Assay	Dilution factor (dry)[a]	Dilution factor (wet)[b]	CORT or GCM as run[c]	ng CORT or GCM as run	CORT spike (ng)[d]	% CORT spike recovery
Feath336pooled_2	336	feather	RIA	33.0	NA	32.18	0.03	NA	NA
Feath336pooled_3	336	feather	RIA	33.0	NA	33.43	0.03	NA	NA
Feath336pooled_1S	336	feather	RIA	33.0	NA	65.96	0.07	0.03	101
Feath336pooled_2S	336	feather	RIA	33.0	NA	64.11	0.06	0.03	96
Feath336pooled_3S	336	feather	RIA	33.0	NA	67.28	0.07	0.03	105
pooledP(stock)_1	pooled	plasma	RIA	NA	1.0	62.07	0.06	NA	NA
pooledP(stock)_2	pooled	plasma	RIA	NA	1.0	65.27	0.07	NA	NA
pooledP(stock)_3	pooled	plasma	RIA	NA	1.0	65.93	0.07	NA	NA
pooledP(stock)_1S	pooled	plasma	RIA	NA	1.0	117.50	0.12	0.05	99
pooledP(stock)_2S	pooled	plasma	RIA	NA	1.0	113.83	0.11	0.05	92
pooledP(stock)_3S	pooled	plasma	RIA	NA	1.0	114.52	0.11	0.05	93
650 #4	650	urates	RIA	0.1	0.5	25.24	0.03	NA	NA
650 #4b	650	urates	RIA	0.0	0.3	14.26	0.01	NA	NA
650 #4c	650	urates	RIA	0.0	0.1	7.54	0.01	NA	NA
650 #4s	650	urates	RIA	0.1	0.5	76.46	0.08	0.05	102
650 #4bs	650	urates	RIA	0.0	0.3	36.88	0.04	0.03	90
650 #4cs	650	urates	RIA	0.0	0.1	18.48	0.02	0.01	88
448 #3_1	448	urates	RIA	0.0	2.2	88.01	0.09	NA	NA
448 #3_2	448	urates	RIA	0.0	2.2	86.95	0.09	NA	NA
448 #3_3	448	urates	RIA	0.0	2.2	88.74	0.09	NA	NA
448 #3_1S	448	urates	RIA	0.0	2.2	177.91	0.18	0.08	108
448 #3_2S	448	urates	RIA	0.0	2.2	178.08	0.18	0.08	108
448 #3_3S	448	urates	RIA	0.0	2.2	179.20	0.18	0.08	110

a. For feather and urates: mg sample dry/100mL assay buffer
b. For urates: mg sample wet/100mL assay buffer; for plasma: µL sample /100mL assay buffer
c. Plasma CORT, feather CORT, or urate GCM concentration as run (pg/tube for RIA, ng/mL assay buffer for ELISA)
d. Exogenous corticosterone spike in ng as run.
