# Supplementary material for: Glucocorticoid measurement in plasma, urates, and feathers from California condors (Gymnogyps californianus) in response to a human-induced stressor
Source: PLoS One. 2018 Oct 23;13(10):e0205565. doi: 10.1371/journal.pone.0205565 (PMC6198957; doi:10.1371/journal.pone.0205565)
Supplement: S5 Table — a. For feather and urates: mg sample dry/100mL assay buffer b. For urates: mg sample wet/100mL assay buffer; for plasma: μL sample /100mL assay buffer c. Plasma CORT, feather CORT, or urate GCM concentration as run (ng hormone/mL assay buffer) d. Plasma CORT or urate GCM concentration in wet sample (ng hormone/mL plasma or ng hormone/g urates wet wt.) e. Feather CORT, or urate GCM concentration in dry sample (ng hormone/g dry wt. for urates and feather) f. Total ng CORT or GCM in sample. (PDF) [file pone.0205565.s012.pdf]

S5 Table. Samples used in RIA vs. ELISA method comparison

Condor ID	# in series	Sample Type	Wet Mass (g)	Dry Mass (g)	RIA Dilution factor (dry)[a]	RIA Dilution factor (wet)[b]	RIA CORT or GCM as run[c]	RIA CORT or GCM wet sample[d]	RIA CORT or GCM dry sample[e]	RIA Total CORT or GCM (ng)[f]	ELISA Dilution factor (dry)[a]	ELISA Dilution factor (wet)[b]	ELISA CORT or GCM as run[c]	ELISA CORT or GCM wet sample[d]	ELISA CORT or GCM dry sample[e]	ELISA Total CORT or GCM (ng)[f]
312	312_6	Feather	NA	0.017	1.86	NA	0.5	NA	28	0.47	1.12	NA	0.2	NA	20	0.33
312	312_7	Feather	NA	0.027	4.50	NA	0.3	NA	11	0.30	1.80	NA	0.2	NA	13	0.35
312	312_9	Feather	NA	0.037	4.15	NA	0.4	NA	9	0.34	2.49	NA	0.2	NA	7	0.27
312	312_12	Feather	NA	0.036	4.04	NA	0.5	NA	34	1.22	2.42	NA	0.2	NA	9	0.33
336	336_4	Feather	NA	0.040	4.42	NA	0.9	NA	20	0.81	2.65	NA	0.4	NA	14	0.57
336	336_6	Feather	NA	0.062	6.89	NA	0.4	NA	7	0.40	4.14	NA	0.2	NA	6	0.36
336	336_10	Feather	NA	0.064	7.09	NA	0.4	NA	6	0.37	4.25	NA	0.3	NA	7	0.45
606	NA	Plasma	NA	NA	NA	1.0	0.4	42	NA	NA	NA	2.5	1.0	39	NA	NA
615	NA	Plasma	NA	NA	NA	1.0	0.5	49	NA	NA	NA	2.5	0.8	33	NA	NA
626	NA	Plasma	NA	NA	NA	1.0	0.6	63	NA	NA	NA	2.5	0.9	38	NA	NA
631	NA	Plasma	NA	NA	NA	1.0	0.3	26	NA	NA	NA	2.5	0.6	24	NA	NA
646	NA	Plasma	NA	NA	NA	1.0	0.6	59	NA	NA	NA	2.5	1.1	45	NA	NA
650	NA	Plasma	NA	NA	NA	0.5	0.4	71	NA	NA	NA	2.5	1.8	71	NA	NA
652	NA	Plasma	NA	NA	NA	0.5	0.4	77	NA	NA	NA	2.5	1.6	62	NA	NA
631	NA	Plasma	NA	NA	NA	1.0	0.3	26	NA	NA	NA	2.5	0.6	24	NA	NA
646	NA	Plasma	NA	NA	NA	1.0	0.6	59	NA	NA	NA	2.5	1.1	45	NA	NA
650	NA	Plasma	NA	NA	NA	0.5	0.4	71	NA	NA	NA	2.5	1.8	71	NA	NA
652	NA	Plasma	NA	NA	NA	0.5	0.4	77	NA	NA	NA	2.5	1.6	62	NA	NA
231	#1	Urates	3.303	0.164	0.16	3.3	2.1	62	1248	205	0.41	8.3	2.6	32	640	104.98
231	#7	Urates	0.985	0.105	0.16	1.5	4.9	316	2946	311	0.40	3.7	1.9	51	474	50.05
448	#1	Urates	2.554	0.126	0.17	3.4	0.9	25	512	65	0.42	8.5	1.4	16	327	41.30
448	#2	Urates	1.413	0.225	0.03	0.2	0.2	114	720	162	0.40	2.5	0.9	38	238	53.40
448	#5	Urates	2.405	0.096	0.08	2.0	0.8	38	946	91	0.21	5.2	0.6	12	290	27.75
448	#6	Urates	1.328	0.047	0.05	1.3	1.1	76	2165	101	0.12	3.3	0.8	23	646	30.15
448	#7	Urates	2.949	0.165	0.04	0.7	0.4	60	1070	176	0.09	1.5	0.4	23	414	68.08
448	1B	Urates	2.250	0.087	0.15	3.8	0.8	19	501	44	0.37	9.6	1.2	12	322	28.09

Condor ID	# in series	Sample Type	Wet Mass (g)	Dry Mass (g)	RIA Dilution factor (dry)[a]	RIA Dilution factor (wet)[b]	RIA CORT or GCM as run[c]	RIA CORT or GCM wet sample[d]	RIA CORT or GCM dry sample[e]	RIA Total CORT or GCM (ng)[f]	ELISA Dilution factor (dry)[a]	ELISA Dilution factor (wet)[b]	ELISA CORT or GCM as run[c]	ELISA CORT or GCM wet sample[d]	ELISA CORT or GCM dry sample[e]	ELISA Total CORT or GCM (ng)[f]
615	#8	Urates	0.112	0.012	0.40	4	1.7	47	433	5	0.05	0.4	0.2	36	331	4.01
626	#1	Urates	1.906	0.054	0.56	20	2.9	14	2270	27	0.27	9.5	0.7	7	245	13.13
626	#10	Urates	1.698	0.102	0.04	0.6	0.4	69	1153	117	0.10	1.6	1.1	69	1159	117.76
631	#9	Urates	0.885	0.030	0.06	1.8	0.6	30	875	26	0.02	0.4	0.7	16	470	14.18
646	n/a	Urates	0.096	0.059	0.12	0.2	0.3	171	280	16	0.29	0.5	0.3	53	86	5.04
650	#1	Urates	0.228	0.045	1.87	10	1.5	16	300	4	0.22	1.1	0.1	10	52	2.32
650	#4	Urates	6.694	0.680	0.05	0.5	0.3	48	470	320	0.20	2.0	0.8	42	412	280.15
652	#4	Urates	2.513	0.276	0.05	0.5	1.2	246	2242	619	0.20	1.8	1.2	69	626	172.92
626	#10	Urates	1.698	0.102	0.04	0.6	0.4	69	1153	117	0.10	1.6	1.1	69	1159	117.76
631	#9	Urates	0.885	0.030	0.06	1.8	0.6	30	875	26	0.02	0.4	0.7	16	470	14.18
646	n/a	Urates	0.096	0.059	0.12	0.2	0.3	171	280	16	0.29	0.5	0.3	53	86	5.04
650	#1	Urates	0.228	0.045	1.87	10	1.5	16	300	4	0.22	1.1	0.1	10	52	2.32
650	#4	Urates	6.694	0.680	0.05	0.5	0.3	48	470	320	0.20	2.0	0.8	42	412	280.15
652	#4	Urates	2.513	0.276	0.05	0.5	1.2	246	2242	619	0.20	1.8	1.2	69	626	172.92

a. For feather and urates: mg sample dry/100mL assay buffer

b. For urates: mg sample wet/100mL assay buffer; for plasma: µL sample /100mL assay buffer

c. Plasma CORT, feather CORT, or urate GCM concentration as run (ng hormone/mL assay buffer)

d. Plasma CORT or urate GCM concentration in wet sample (ng hormone/mL plasma or ng hormone/g urates wet wt.)

e. Feather CORT, or urate GCM concentration in dry sample (ng hormone/g dry wt. for urates and feather)

f. Total ng CORT or GCM in sample
