# Supplementary material for: Glucocorticoid measurement in plasma, urates, and feathers from California condors (Gymnogyps californianus) in response to a human-induced stressor
Source: PLoS One. 2018 Oct 23;13(10):e0205565. doi: 10.1371/journal.pone.0205565 (PMC6198957; doi:10.1371/journal.pone.0205565)
Supplement: S6 Table — a. Time of sample collection as hours since bird was trapped from the wild. Condors are caught and moved into flight pen using a double door trap operated from a blind, and therefor do not see a human until the flight pen entry by technicians on handling days. b. Time of sample collection as minutes since initial flight pen entry by technicians. This precedes handling start. c. Time of sample collection as minutes since handling start. Handling start was recorded when condor was trapped in hoop net. (PDF) [file pone.0205565.s013.pdf]

S6 Table. Collection and CORT data for plasma samples

Condor ID	Date Coll.	Time since trapped from wild[a] (hr)	Time since initial pen entry[b] (hr)	Time since handling[c] (min)	CORT (ng/mL)
23	6/14/2016	n/a	155	4	58
120	6/14/2016	n/a	n/a	n/a	47
159	6/14/2016	n/a	135	9	47
174	7/28/2016	n/a	10	8	22
199	6/3/2015	23	200	5	107
204	6/3/2015	28	43	6	116
209	10/29/2015	27	65	6	189
340	10/14/2015	43	34	4	77
351	6/10/2015	19	45	4	80
448	6/16/2014	20	14	3	73
463	10/28/2015	46	50	5	141
464	7/28/2016	n/a	109	9	105
470	10/29/2015	21	31	6	189
477	5/28/2015	27	58	7	68
538	5/27/2015	92	14	9	70
544	7/28/2016	n/a	73	8	87
547	6/3/2015	n/a	97	4	112
564	6/3/2015	n/a	128	9	49
567	5/28/2015	223	177	11	139
583	5/6/2015	n/a	42	13	131
597	10/7/2015	118	80	5	118
603	7/28/2016	n/a	141	9	1
606	10/21/2015	72	60	18	60
615	6/4/2014	118	30	9	49
626	10/29/2014	48	20	6	63
631	1/7/2014	n/a	61	10	26
631	10/14/2015	141	12	5	31
646	1/7/2014	n/a	81	10	59
650	1/7/2014	n/a	26	10	71
650	10/14/2015	144	174	5	87
652	1/7/2014	n/a	41	4	77
663	5/28/2015	30	127	6	82
684	10/14/2015	47	137	6	66
687	10/7/2015	45	43	7	51
688	6/3/2015	n/a	155	4	13
692	6/10/2015	19	13	5	16
700	10/7/2015	119	18	5	40
704	10/21/2015	23	132	7	62
729	10/14/2015	120	73	5	92
745	5/28/2015	n/a	33	13	83

Condor ID	Date Coll.	Time since trapped from wild[a] (hr)	Time since initial pen entry[b] (hr)	Time since handling[c] (min)	CORT (ng/mL)
769	6/3/2015	26	74	6	55

a. Time of sample collection as hours since bird was trapped from the wild. Condors are caught and moved into flight pen using a double door trap operated from a blind, and therefor do not see a human until the flight pen entry by technicians on handling days.
b. Time of sample collection as minutes since initial flight pen entry by technicians. This precedes handling start.
c. Time of sample collection as minutes since handling start. Handling start was recorded when condor was trapped in hoop net.
