# Supplementary material for: Glucocorticoid measurement in plasma, urates, and feathers from California condors (Gymnogyps californianus) in response to a human-induced stressor
Source: PLoS One. 2018 Oct 23;13(10):e0205565. doi: 10.1371/journal.pone.0205565 (PMC6198957; doi:10.1371/journal.pone.0205565)
Supplement: S7 Table — a. Time of sample collection as hours since bird was trapped from the wild. Condors are caught and moved into flight pen using a double door trap operated from a blind, and therefor do not see a human until the flight pen entry by technicians on handling days. b. Time of sample collection as minutes since initial flight pen entry by technicians. This precedes handling start. c. Time of sample collection as minutes since handling start. Handling start was recorded when condor was trapped in hoop net. (PDF) [file pone.0205565.s014.pdf]

S7 Table. Collection and GCM data for urate samples

Condor ID	# in series	Date Collected	Wet mass (g)	Dry mass (g)	GCM (ng/g wet)	GCM (ng/g dry)	Total GCM (ng)	Time since trapped from wild [a] (hr)	Time since initial pen entry[b] (min)	Time since handling[c] (min)
23	1	6/14/2016	5.141	0.297	36	620	190	NA	173	22
23	2	6/14/2016	0.396	0.025	31	480	12	NA	190	39
23	3	6/14/2016	0.116	0.016	83	620	9.6	NA	208	57
23	4	6/14/2016	0.224	0.020	110	1200	24	NA	217	66
23	5	6/14/2016	0.147	0.013	110	1200	16	NA	234	83
23	6	6/14/2016	0.241	0.033	120	860	29	NA	269	118
23	7	6/14/2016	0.464	0.061	140	1100	67	NA	287	136
23	8	6/14/2016	0.114	0.017	120	770	13	NA	306	155
23	9	6/14/2016	0.568	0.064	150	1400	88	NA	338	187
23	10	6/14/2016	0.109	0.014	130	1000	14	NA	365	214
23	12	6/14/2016	1.568	0.199	110	860	170	NA	387	236
120	1	6/14/2016	11.485	0.201	7.7	440	89	NA	96	28
120	2	6/14/2016	1.484	0.035	8.1	340	12	NA	104	36
120	3	6/14/2016	2.647	0.332	59	470	160	NA	127	59
120	4	6/14/2016	1.923	0.206	37	340	70	NA	139	71
120	5	6/14/2016	3.928	0.246	25	400	98	NA	154	86
120	6	6/14/2016	2.196	0.115	25	480	55	NA	159	91
120	7	6/14/2016	1.946	0.081	25	590	48	NA	164	96
120	8	6/14/2016	4.068	0.134	17	510	68	NA	166	98
120	9	6/14/2016	1.916	0.059	22	720	42	NA	181	113
120	10	6/14/2016	4.167	0.178	24	570	100	NA	193	125
120	11	6/14/2016	3.010	0.172	31	540	93	NA	208	140
120	12	6/14/2016	2.153	0.108	26	510	55	NA	225	157
120	13	6/14/2016	6.853	0.277	25	620	170	NA	238	170
120	14	6/14/2016	0.027	0.005	18	100	0.5	NA	245	177
120	15	6/14/2016	6.230	0.472	36	470	220	NA	254	186
120	16	6/14/2016	4.688	0.370	32	410	150	NA	265	197
120	17	6/14/2016	5.022	0.500	52	530	260	NA	283	215
120	18	6/14/2016	2.653	0.251	47	500	130	NA	302	234
120	19	6/14/2016	5.270	0.247	36	780	190	NA	306	238
120	20	6/14/2016	1.690	0.138	16	200	27	NA	328	260
120	21	6/14/2016	2.263	0.158	43	620	97	NA	341	273
120	22	6/14/2016	1.649	0.122	59	800	97	NA	358	290
120	23	6/14/2016	0.458	0.034	80	1100	37	NA	387	319
120	25	6/14/2016	2.371	0.088	51	1400	120	NA	404	336
159	1	6/14/2016	2.481	0.566	6	26	15	NA	148	22
159	2	6/14/2016	0.713	0.206	32	110	23	NA	164	38
159	4+5	6/14/2016	3.565	0.799	34	150	120	NA	183	57

Condor ID	# in series	Date Collected	Wet mass (g)	Dry mass (g)	GCM (ng/g wet)	GCM (ng/g dry)	Total GCM (ng)	Time since trapped from wild [a] (hr)	Time since initial pen entry[b] (min)	Time since handling[c] (min)
159	6	6/14/2016	0.798	0.109	34	250	27	NA	225	99
159	7	6/14/2016	0.967	0.121	32	260	31	NA	238	112
159	9	6/14/2016	1.043	0.087	23	280	24	NA	249	123
159	10	6/14/2016	5.025	0.280	16	290	81	NA	268	142
159	12	6/14/2016	1.224	0.060	9.8	200	12	NA	288	162
159	13	6/14/2016	8.079	0.816	16	160	130	NA	296	170
159	14	6/14/2016	5.108	0.389	25	330	130	NA	312	186
159	15	6/14/2016	4.742	0.474	36	360	170	NA	344	218
159	17	6/14/2016	0.643	0.045	35	500	22	NA	362	236
159	20	6/14/2016	2.847	0.221	47	610	130	NA	391	265
159	22	6/14/2016	0.107	0.023	64	290	6.8	NA	410	284
174	1	7/28/2016	1.323	0.124	53	560	70	NA	27	25
174	3	7/28/2016	0.402	0.050	100	840	42	NA	84	82
174	4	7/28/2016	2.532	0.151	63	1100	160	NA	116	114
174	5	7/28/2016	0.815	0.054	51	770	42	NA	143	141
174	6	7/28/2016	0.200	0.024	63	530	13	NA	170	168
174	7	7/28/2016	1.278	0.174	170	1200	210	NA	219	217
174	9	7/28/2016	0.982	0.167	130	760	130	NA	281	279
174	11	7/28/2016	0.075	0.016	0.8	4	0.1	NA	284	282
209	1	10/29/2015	3.561	0.175	75	1500	270	28.0	98	39
209	2	10/29/2015	0.823	0.042	100	2000	84	28.1	105	46
209	3	10/29/2015	0.667	0.046	130	1900	86	28.3	115	56
209	4	10/29/2015	0.127	0.022	160	900	20	28.4	123	64
209	5	10/29/2015	0.639	0.067	240	2300	150	28.6	132	73
209	6	10/29/2015	0.045	0.013	190	670	8.7	28.8	148	89
209	7	10/29/2015	0.172	0.016	94	1000	16	29.1	162	103
209	8	10/29/2015	1.153	0.038	95	2900	110	29.2	171	112
340	1	10/14/2015	2.053	0.033	7.5	470	15	43.2	57	27
340	3	10/14/2015	2.321	0.069	16	530	37	43.3	62	32
340	4	10/14/2015	3.682	0.063	14	840	53	43.4	66	36
340	6	10/14/2015	2.473	0.047	24	1300	60	43.5	73	43
340	7	10/14/2015	0.069	0.011	60	360	4.1	43.7	86	56
340	8	10/14/2015	0.139	0.015	100	960	14	43.8	91	61
340	9	10/14/2015	0.230	0.028	210	1700	48	44.1	108	78
340	10	10/14/2015	0.044	0.016	180	490	8	44.1	111	81
340	11	10/14/2015	0.265	0.034	310	2400	81	44.3	119	89
340	12	10/14/2015	0.129	0.017	290	2200	38	44.4	128	98
340	13	10/14/2015	0.143	0.024	320	1900	45	44.9	155	125
340	14	10/14/2015	0.422	0.031	240	3300	100	44.9	157	127
340	15	10/14/2015	0.261	0.015	230	4000	61	45.2	172	142

Condor ID	# in series	Date Collected	Wet mass (g)	Dry mass (g)	GCM (ng/g wet)	GCM (ng/g dry)	Total GCM (ng)	Time since trapped from wild [a] (hr)	Time since initial pen entry[b] (min)	Time since handling[c] (min)
340	16	10/14/2015	0.315	0.030	390	4100	120	45.5	192	162
340	17	10/14/2015	0.223	0.020	420	4600	94	45.6	201	171
351	1	6/10/2015	0.316	0.029	61	670	19	19.1	76	35
351	2	6/10/2015	2.729	0.079	26	880	70	19.2	82	41
351	4	6/10/2015	0.702	0.047	21	310	15	19.3	92	51
448	1	6/16/2014	4.804	0.126	25	510	120	20.2	40	29
448	2	6/16/2014	1.413	0.225	110	720	160	20.8	78	67
448	3	6/16/2014	4.732	0.295	40	3800	190	21.4	110	99
448	4	6/16/2014	0.961	0.071	76	1000	73	21.6	122	111
448	5	6/16/2014	2.405	0.096	38	950	91	21.8	138	127
448	6	6/16/2014	1.328	0.047	76	2200	100	22.5	178	167
448	7	6/16/2014	2.949	0.165	36	640	110	22.7	188	177
463	1	10/28/2015	7.668	0.061	4.6	580	35	22.2	76	31
463	2	10/28/2015	1.188	0.057	22	470	26	22.2	79	34
463	3	10/28/2015	0.309	0.035	65	560	20	22.3	84	39
463	4	10/28/2015	0.260	0.034	64	490	17	22.3	86	41
463	5	10/28/2015	0.189	0.026	75	550	14	22.4	88	43
463	6	10/28/2015	0.272	0.032	96	820	26	22.4	90	45
463	8	10/28/2015	0.131	0.015	110	940	14	22.4	93	48
463	10	10/28/2015	0.820	0.095	160	1400	130	22.6	102	57
464	1	7/28/2016	0.362	0.023	91	1400	33	NA	124	24
464	2	7/28/2016	1.058	0.122	180	1600	190	NA	147	47
464	3	7/28/2016	4.224	0.204	55	1100	230	NA	188	88
464	4	7/28/2016	2.296	0.095	53	1300	120	NA	216	116
464	5	7/28/2016	0.072	0.005	0.8	12	0.1	NA	220	120
464	6	7/28/2016	1.358	0.089	300	4600	410	NA	263	163
464	7	7/28/2016	0.747	0.098	260	2000	190	NA	287	187
464	8	7/28/2016	1.698	0.192	270	2400	460	NA	345	245
470	1	10/29/2015	1.116	0.123	29	260	32	21.4	54	29
470	2	10/29/2015	0.499	0.025	100	2100	51	21.6	63	38
470	3	10/29/2015	0.065	0.005	150	1800	9.6	21.7	72	47
470	4	10/29/2015	0.032	0.010	330	1000	11	21.8	78	53
470	5	10/29/2015	0.074	0.011	480	3100	35	22.0	90	65
470	6	10/29/2015	0.165	0.020	610	5100	100	22.2	100	75
470	7	10/29/2015	0.225	0.028	640	5200	140	22.5	120	95
470	8	10/29/2015	0.385	0.050	540	4200	210	22.7	134	109
470	9	10/29/2015	0.497	0.053	700	6600	350	22.9	144	119
470	10	10/29/2015	0.273	0.038	640	4600	180	23.1	154	129
470	11	10/29/2015	0.252	0.032	930	7200	230	23.6	183	158
538	1	5/27/2015	2.286	0.114	42	850	97	92.3	49	44

Condor ID	# in series	Date Collected	Wet mass (g)	Dry mass (g)	GCM (ng/g wet)	GCM (ng/g dry)	Total GCM (ng)	Time since trapped from wild [a] (hr)	Time since initial pen entry[b] (min)	Time since handling[c] (min)
538	2	5/27/2015	0.959	0.123	18	140	17	92.4	52	47
538	4	5/27/2015	0.108	0.007	38	560	4.1	92.5	59	54
538	5	5/27/2015	0.759	0.046	49	810	37	92.5	62	57
538	7	5/27/2015	1.796	0.044	47	1900	85	92.6	64	59
538	8	5/27/2015	1.180	0.032	90	3300	110	92.7	75	70
544	1	7/28/2016	2.545	0.095	18	470	45	NA	92	27
544	2	7/28/2016	1.549	0.164	130	1200	190	NA	146	81
544	3	7/28/2016	2.615	0.349	63	470	160	NA	242	177
544	4	7/28/2016	1.091	0.120	170	1500	180	NA	287	222
544	7	7/28/2016	0.671	0.200	130	430	87	NA	320	255
547	1	6/3/2015	2.979	0.056	14	720	41	27.8	111	18
547	3	6/3/2015	0.928	0.017	20	1100	18	27.9	121	28
547	4	6/3/2015	1.215	0.024	22	1100	27	28.1	133	40
547	5	6/3/2015	0.770	0.017	31	1400	24	28.2	136	43
547	7	6/3/2015	0.785	0.026	56	1700	44	28.7	166	73
547	8	6/3/2015	2.328	0.081	43	1200	100	29.0	186	93
547	9	6/3/2015	1.514	0.040	44	1600	66	29.1	189	96
547	10	6/3/2015	0.625	0.027	46	1100	29	29.4	208	115
547	11	6/3/2015	0.349	0.017	38	810	13	29.4	211	118
547	12	6/3/2015	0.940	0.023	34	1400	32	29.5	214	121
547	14	6/3/2015	0.976	0.038	42	1100	41	29.6	222	129
583	2	5/6/2015	3.135	0.276	80	910	250	24.0	62	33
583	3	5/6/2015	2.241	0.427	230	1200	520	24.7	106	77
583	5	5/6/2015	0.153	0.030	470	2400	71	24.8	111	82
606	1	10/21/2015	3.467	0.092	7.8	300	27	72.7	74	32
606	3	10/21/2015	1.435	0.032	5.9	270	8.5	72.9	85	43
606	4	10/21/2015	0.986	0.026	12	440	11	72.9	88	46
606	5	10/21/2015	0.993	0.019	9.2	480	9.1	73.0	93	51
606	6	10/21/2015	3.703	0.133	18	490	65	73.2	108	66
606	7	10/21/2015	0.701	0.049	68	970	47	73.3	111	69
606	8	10/21/2015	0.198	0.016	51	620	10	73.5	124	82
606	9	10/21/2015	0.418	0.059	74	520	31	73.7	137	95
606	10	10/21/2015	0.303	0.033	75	690	23	73.8	141	99
606	11	10/21/2015	0.315	0.029	100	1100	33	73.9	147	105
606	12	10/21/2015	0.884	0.037	23	550	20	74.2	165	123
606	13	10/21/2015	0.043	0.005	37	360	1.6	74.4	177	135
626	1	10/29/2014	1.906	0.054	14	2300	28	48.1	25	11
626	2	10/29/2014	1.083	0.022	6.8	330	7.4	48.6	55	41
626	3	10/29/2014	1.398	0.052	16	430	22	48.7	64	50
626	4	10/29/2014	0.406	0.025	28	460	12	48.7	65	51

Condor ID	# in series	Date Collected	Wet mass (g)	Dry mass (g)	GCM (ng/g wet)	GCM (ng/g dry)	Total GCM (ng)	Time since trapped from wild [a] (hr)	Time since initial pen entry[b] (min)	Time since handling[c] (min)
626	5	10/29/2014	0.663	0.056	70	830	47	48.9	73	59
626	6	10/29/2014	0.619	0.058	82	880	51	49.1	87	73
626	7	10/29/2014	1.015	0.085	130	1500	130	49.4	107	93
626	8	10/29/2014	0.400	0.033	110	1300	44	49.6	117	103
626	10	10/29/2014	1.698	0.102	41	690	70	49.9	135	121
626	11	10/29/2014	0.217	0.032	280	1900	62	51.8	247	233
626	13	10/29/2014	0.286	0.039	370	2700	100	52.2	274	260
631	1	10/14/2015	27.300	0.288	2.9	270	79	141.2	28	21
631	3	10/14/2015	3.311	0.092	3	110	10	141.4	37	30
631	4	10/14/2015	0.528	0.012	0.023	1	0.012	141.4	39	32
631	5	10/14/2015	15.550	0.404	2.8	110	44	141.5	41	34
631	6	10/14/2015	12.907	0.315	3.2	130	41	141.6	48	41
631	7	10/14/2015	5.686	0.064	5	450	29	141.7	58	51
631	8	10/14/2015	4.526	0.076	8.4	500	38	141.8	60	53
631	9	10/14/2015	2.971	0.052	14	770	40	142	76	69
631	10	10/14/2015	1.048	0.018	12	700	13	142.2	85	78
631	11	10/14/2015	4.708	0.077	9.6	580	45	142.3	90	83
631	12	10/14/2015	5.205	0.080	15	1000	80	142.5	101	94
631	13	10/14/2015	3.399	0.061	19	1100	66	142.6	112	105
631	14	10/14/2015	1.965	0.071	42	1200	83	142.9	129	122
631	15	10/14/2015	2.895	0.066	31	1300	88	143	131	124
631	17	10/14/2015	1.945	0.064	60	1800	120	143.5	165	158
631	18	10/14/2015	0.572	0.031	110	2000	62	144.2	206	199
663	1	5/28/2015	1.851	0.149	24	300	45	30.3	152	31
663	2	5/28/2015	0.732	0.126	97	560	71	30.3	156	35
663	4	5/28/2015	0.886	0.144	200	1200	170	31.0	197	76
663	5	5/28/2015	0.093	0.022	190	800	18	31.1	200	79
684	1	6/23/2015	3.410	0.104	31	1000	100	45.3	105	22
684	2	6/23/2015	0.446	0.027	33	560	15	45.3	109	26
684	3	6/23/2015	2.741	0.181	58	870	160	45.5	117	34
684	4	6/23/2015	0.857	0.074	73	850	63	45.7	128	45
687	2	10/7/2015	9.168	0.283	7	230	65	45.7	76	40
687	3	10/7/2015	9.265	0.159	8.3	480	77	45.8	83	47
687	4	10/7/2015	3.054	0.081	14	530	43	46.0	95	59
687	5	10/7/2015	8.020	0.266	15	470	120	46.1	105	69
687	7	10/7/2015	0.590	0.021	24	660	14	46.3	115	79
692	1	6/10/2015	3.544	0.159	4.9	110	17	19.5	34	26
692	2	6/10/2015	4.995	0.250	26	490	130	19.6	36	28
692	3	6/10/2015	2.129	0.061	4.9	170	11	19.7	42	34
692	4	6/10/2015	10.668	0.471	32	530	340	19.8	52	44

Condor ID	# in series	Date Collected	Wet mass (g)	Dry mass (g)	GCM (ng/g wet)	GCM (ng/g dry)	Total GCM (ng)	Time since trapped from wild [a] (hr)	Time since initial pen entry[b] (min)	Time since handling[c] (min)
692	5	6/10/2015	5.112	0.250	7.1	130	36	19.9	58	50
692	6	6/10/2015	1.556	0.111	45	630	69	20.1	66	58
692	7	6/10/2015	1.018	0.121	20	160	20	20.1	69	61
692	8	6/10/2015	3.033	0.159	10	200	32	20.2	72	64
692	10	6/10/2015	2.973	0.164	55	1000	160	20.4	85	77
700	1	10/7/2015	2.434	0.055	8.1	360	20	119.5	35	22
700	2	10/7/2015	1.638	0.024	0.028	1.9	0.046	119.5	38	25
700	3	10/7/2015	3.391	0.073	7.7	360	26	119.6	41	28
700	4	10/7/2015	3.663	0.109	8	270	29	119.6	46	33
700	6	10/7/2015	0.663	0.064	41	420	27	120.1	71	58
700	7	10/7/2015	0.896	0.066	50	680	45	120.2	78	65
700	8	10/7/2015	1.435	0.089	84	1300	120	120.6	102	89
745	1	5/28/2015	11.665	0.162	7.2	520	84	28.5	47	27
745	2	5/28/2015	2.236	0.058	24	910	53	28.8	62	42
745	4	5/28/2015	1.397	0.085	63	1000	87	29.2	86	66
745	5	5/28/2015	0.894	0.047	60	1200	54	29.4	100	80
745	6	5/28/2015	0.201	0.018	53	580	11	29.6	109	89
745	7	5/28/2015	1.089	0.201	110	590	120	30.1	139	119

a. Time of sample collection as hours since bird was trapped from the wild. Condors are caught and moved into flight pen using a double door trap operated from a blind, and therefor do not see a human until the flight pen entry by technicians on handling days.
b. Time of sample collection as minutes since initial flight pen entry by technicians. This precedes handling start.
c. Time of sample collection as minutes since handling start. Handling start was recorded when condor was trapped in hoop net.
