# Supplementary material for: Glucocorticoid measurement in plasma, urates, and feathers from California condors (Gymnogyps californianus) in response to a human-induced stressor
Source: PLoS One. 2018 Oct 23;13(10):e0205565. doi: 10.1371/journal.pone.0205565 (PMC6198957; doi:10.1371/journal.pone.0205565)
Supplement: S8 Table — a. Number of competitive models (listed in Table 2) including the parameter. b. Summed Akaike weights for all models with parameter. c. Weighted average beta coefficient. d. Model averaged standard error. e. 90% confidence interval for parameter estimate. (PDF) [file pone.0205565.s015.pdf]

S8 Table. Multiple linear regression model averaged parameter estimates for plasma CORT levels.

Parameter	*N-models*[a]	*Sum wt.*[b]	Estimate[c]	SE[d]	90% CI[e] Upper	Lower
Intercept	--	--	47.00	15.69	72.73	21.27
Age	27	1.00	72.64	21.40	107.74	37.54
Season	9	0.34	9.83	7.42	22.00	-2.33
Keel	8	0.24	-10.32	10.71	7.24	-27.88
Hydration	7	0.21	7.99	7.50	20.29	-4.30
Min Since Handling	7	0.17	9.31	13.73	31.82	-13.21
Sex	6	0.14	-3.53	9.08	11.36	-18.41
Hr Since Trapped	5	0.12	6.49	15.26	31.51	-18.54
Min Since Entry	4	0.11	7.42	13.39	29.39	-14.54

[a]Number of competitive models (listed in Table 2) including the parameter. [b]Summed Akaike weights for all models with parameter. [c]Weighted average beta coefficient. [d]Model averaged standard error. [e]90% confidence interval for parameter estimate.
