# Supplementary material for: Glucocorticoid measurement in plasma, urates, and feathers from California condors (Gymnogyps californianus) in response to a human-induced stressor
Source: PLoS One. 2018 Oct 23;13(10):e0205565. doi: 10.1371/journal.pone.0205565 (PMC6198957; doi:10.1371/journal.pone.0205565)
Supplement: S9 Table — a. Number of competitive models (listed in Table 2) including the parameter. b. Summed Akaike weights for all models with parameter. c. Weighted average beta coefficient. d. Model averaged standard error. e. 90% confidence interval for parameter estimate. (PDF) [file pone.0205565.s016.pdf]

S9 Table. Multiple linear regression model averaged parameter estimates for 1st urate GCM.

Parameter	*N-models*[a]	*Sum wt.*[b]	Estimate[c]	SE[d]	90% CI[e] Upper	Lower
Intercept	--	--	171	153	422	-80.1
Age	13	0.79	764	285	1230	297
Sex	8	0.66	-155	68.2	-43.5	-267
Plasma CORT	9	0.45	304	157	561	47.5
Season	5	0.27	102	63.6	206	-2.03
Min Since Handling	3	0.11	167	132	384	-50.0
Min Since Entry	2	0.09	217	225	586	-153
Hr Since Trapped	2	0.05	51.2	185	354	-252

[a]Number of competitive models (listed in Table 2) including the parameter. [b]Summed Akaike weights for all models with parameter. [c]Weighted average beta coefficient. [d]Model averaged standard error. [e]90% confidence interval for parameter estimate.
